# The Functional Interplay Between the t(9;22)-Associated Fusion Proteins BCR/ABL and ABL/BCR in Philadelphia Chromosome-Positive Acute Lymphatic Leukemia

**DOI:** 10.1371/journal.pgen.1005144

**Published:** 2015-04-28

**Authors:** Anahita Rafiei, Afsar Ali Mian, Claudia Döring, Anna Metodieva, Claudia Oancea, Frederic B. Thalheimer, Martin Leo Hansmann, Oliver Gerhard Ottmann, Martin Ruthardt

**Affiliations:** 1 Department of Hematology, Goethe University Hospital, Frankfurt, Germany; 2 Dr. Senckenberg Institute of Pathology, Goethe University Hospital, Frankfurt, Germany; Fred Hutchinson Cancer Research Center, UNITED STATES

## Abstract

The hallmark of Philadelphia chromosome positive (Ph^+^) leukemia is the BCR/ABL kinase, which is successfully targeted by selective ATP competitors. However, inhibition of BCR/ABL alone is unable to eradicate Ph^+^ leukemia. The t(9;22) is a reciprocal translocation which encodes not only for the der22 (Philadelphia chromosome) related BCR/ABL, but also for der9 related ABL/BCR fusion proteins, which can be detected in 65% of patients with chronic myeloid leukemia (CML) and 100% of patients with Ph^+^ acute lymphatic leukemia (ALL). ABL/BCRs are oncogenes able to influence the lineage commitment of hematopoietic progenitors. Aim of this study was to further disclose the role of p96^*ABL/BCR*^ for the pathogenesis of Ph^+^ ALL. The co-expression of p96^*ABL/BCR*^ enhanced the kinase activity and as a consequence, the transformation potential of p185^*BCR/ABL*^. Targeting p96^*ABL/BCR*^ by RNAi inhibited growth of Ph^+^ ALL cell lines and Ph^+^ ALL patient-derived long-term cultures (PD-LTCs). Our *in vitro* and *in vivo* stem cell studies further revealed a functional hierarchy of p96^*ABL/BCR*^ and p185^*BCR/ABL*^ in hematopoietic stem cells. Co-expression of p96^*ABL/BCR*^ abolished the capacity of p185^*BCR/ABL*^ to induce a CML-like disease and led to the induction of ALL. Taken together our here presented data reveal an important role of p96^*ABL/BCR*^ for the pathogenesis of Ph^+^ ALL.

## Author Summary

The t(9;22) is a reciprocal translocation, which causes chronic myeloid leukemia (CML) and a subset of high risk acute lymphatic leukemia (ALL). The derivative chromosome 22 is the so called Philadelphia chromosome (Ph) which encodes the BCR/ABL kinase. Targeting BCR/ABL by selective ATP competitors, such as imatinib or nilotinib, is a well validated therapeutic concept, but unable to definitively eradicate the disease. Little is known about the role of the fusion protein encoded by the reciprocal derivative chromosome 9, the ABL/BCR. In models of Ph^+^ ALL we show that the functional interplay between ABL/BCR and BCR/ABL not only increases the transformation potential of BCR/ABL but is also indispensable for the growth and survival of Ph^+^ ALL leukemic cells. The presence of ABL/BCR changed the phenotype of the leukemia most likely due to its capacity to influence the stem cell population as shown by our *in vivo* data. Taken together our here presented data reveal an important role of p96^*ABL/BCR*^ for the pathogenesis of Ph^+^ ALL.

## Introduction

The Philadelphia chromosome (Ph) is the cytogenetic correlate of der22 formed by the t(9;22)(q34;q11). The t(9;22) is a reciprocal translocation [1]. Two principal breaks occur in the *BCR* (breakpoint cluster region) gene locus on chromosome 22: the (major) M-BCR, between exons 12 and 16, and the (minor) m-BCR, in the first intron of *BCR*. On der22, M-BCR leads to the creation of p210^*BCR/ABL*^ and m-BCR to that of p185^*BCR/ABL*^. The breakpoint in the *ABL* (Abelson tyrosin protein kinase 1) gene on chromosome 9 falls within the intron between the exons 1 and 2. Therefore the ABL-part of the t(9;22) fusion proteins is constant [1].

The breakpoint on der22 is decisive for the determination of the phenotype of the Ph^+^ leukemias. In fact M-BCR p210^*BCR/ABL*^ is associated with primarily myeloid leukemia. p210^*BCR/ABL*^ is pathognomonic for the chronic myeloid leukemia (CML). In the very rare cases of Ph^+^ acute myeloid leukemia (AML) the great majority of the patients harbors the p210^*BCR/ABL*^ [2,3]. In contrast, p185^*BCR/ABL*^ is nearly exclusively detected in Ph^+^ acute lymphatic leukemia (ALL) [4].

On the other hand about 30% of patients with Ph^+^ ALL harbor the M-BCR p210^*BCR/ABL*^. There are differences regarding the prognosis between Ph^+^ ALL patients harboring either the p185^*BCR/ABL*^ or p210^*BCR/ABL*^ [5,6,7]. Furthermore the progression of chronic phase (CP) CML, if untreated, leads in most of the cases to a myeloid blast crisis (BC). Only 30% of patients develop lymphatic BC [1,8,9]. The development of lymphatic BC has been attributed to an increased kinase activity of p210^*BCR/ABL*^ as compared to patients with myeloid BC [10]. Factors able to modify the kinase activity of p210^*BCR/ABL*^ are still completely unknown.

The fusion proteins p185^*BCR/ABL*^ and p210^*BCR/ABL*^ are mutant ABL kinases. The native ABL kinase is finely regulated in response to growth factors and other stimuli [11]. Through the fusion to BCR, ABL constitutively activates its “down-stream” signaling pathways, including RAS, JAK/STAT and PI-3 kinase [1,12]. In primary murine hematopoietic models we have shown that both p210^*BCR/ABL*^ and p185^*BCR/ABL*^ allow only a myeloid commitment/differentiation of hematopoietic stem cells (HSCs). Both suppress the lymphatic commitment of HSCs by the suppression of the B-cell signaling [13]. Even on leukemic blasts of patients with Ph^+^ ALL there is a high frequency of myeloid marker expression such as CD33 and CD13 [14]. The inhibitory effect of BCR/ABL on the B-cell signaling is counteracted by the ABL/BCRs, the reciprocal t(9;22) fusion proteins [13].

The *ABL/BCR* fusion gene on der9 differs between m-BCR-p185^*BCR/ABL*^ and M-BCR-p210^*BCR/ABL*^. In the case of M-BCR the reciprocal *ABL/BCR* gene encodes a “small” ABL/BCR with an approximate molecular mass of 40 kDa (p40^*ABL/BCR*^)[13], whereas in the case of m-BCR it encodes a “larger” ABL/BCR of 96 kDa (p96^*ABL/BCR*^)[13]. The p40^*ABL/BCR*^ transcript is detectable in 65% of the CML patients [15] and the p96^*ABL/BCR*^ transcript is present in 100% of examined patients with m-BCR Ph^+^ ALL [16].

The p96^*ABL/BCR*^ and p40^*ABL/BCR*^ are BCR mutants [17]. Native BCR acts as a negative regulator of proliferation and oncogenic transformation by a down-regulation of RAS-mediated signaling [18]. Furthermore, it inhibits Wnt signaling by blocking TCF-1/β-catenin-mediated transcription [19]. BCR harbors both RHO-GEF and RAC-GAP functions and controls cytoskeleton modeling by regulating the activity of RHO-like GTPases [17,20]. Most likely by the loss of regulatory domains both p96^*ABL/BCR*^ and p40^*ABL/BCR*^ activate RAC, a key player in the leukemogenesis of Ph^+^ leukemia [21], which contributes to their leukemogenic potential *in vivo* and increases the proliferation of the stem cell capacity of murine HSC [13].

The aim of this study was to disclose the functional interplay of p96^*ABL/BCR*^ and p185^*BCR/ABL*^ in the induction and maintenance of Ph^+^ ALL. Therefore we studied the role of p96^*ABL/BCR*^ for i.) the transformation potential of p185^*BCR/ABL*^; ii.) the survival of Ph^+^ ALL cells; iii.) the stem cell capacity of p185^*BCR/ABL*^-positive HSCs; iv.) the drug response of Ph^+^ ALL cells and v.) the lineage commitment of HSCs *in vivo*.

## Results

### Co-expression of p96^*ABL/BCR*^ enhances the proliferation and transformation potential of p185^*BCR/ABL*^ accompanied by enhanced kinase activity of p185^*BCR/ABL*^ and increased MAP-kinase signaling

**Fig 1:**
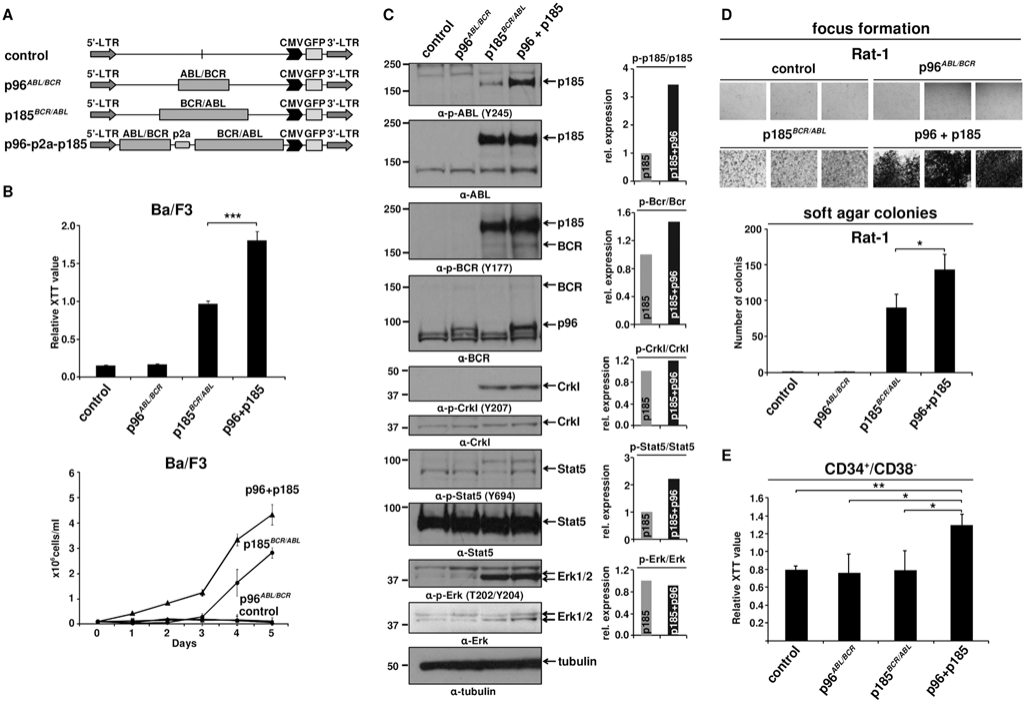
Effect of p96^*ABL/BCR*^ on the proliferation and transformation capacity of p185^*BCR/ABL*^-positive cells. (A) Schematic representation of proviruses encoding the transgenes used in this experiment; (B) IL-3 independent growth of Ba/F3 cells expressing the indicated fusion proteins. Proliferation was measured using XTT-assay and growth by dye exclusion using trypan blue. The results are given as means of 3 independent experiments ± SD; (C) Detection of the expression of transgenes in Ba/F3 cells and activation/phosphorylation status of Crkl, Stat5 and Erk1/2 by immunoblotting using the indicated antibodies. The bar graphs represent the relative quantification of phosphorylated with respect to total protein of p185^*BCR/ABL*^ (p185) and p96^*ABL/BCR*^-p185^*BCR/ABL*^ co-expressing cells (p96 + p185); (D) Transformation assays in Rat-1 cells. For focus formation retrovirally transduced Rat-1 cells were incubated for 15 days in 24-well plates. All experiments were performed at least three times with similar results. For colony formation assay seeded at 5x10^3^ cells/well in soft-agar in 6-well-plates. After 15 days, the colonies were counted. The mean of three independent experiments each performed in triplicates ± SD is given; (E) Effect of co-expression of p96^*ABL/BCR*^ and p185^*BCR/ABL*^ on the proliferation of human HSCs. Proliferation of lentivirally transduced CD34^+^/CD38^-^ HSCs was measured using XTT-assay. The mean of three independent experiments is given ± SD.

All p185^*BCR/ABL*^-positive Ph^+^ ALL patients express p96^*ABL/BCR*^ at both mRNA and protein levels [13,16], but experimental models only focus on the role of p185^*BCR/ABL*^ alone. Thus, we investigated the effect of p96^*ABL/BCR*^ on the capacity of p185^*BCR/ABL*^ to confer factor independent growth in Ba/F3 cells. Co-expression of p96^*ABL/BCR*^ and p185^*BCR/ABL*^ in Ba/F3 cells was achieved by expressing the two transgenes from a p2a peptide-linked multi-cistronic retroviral vector (Fig 1A). The expression of the transgenes was further confirmed by immunoblotting in transduced Ba/F3 cells (Fig 1C). Proliferation and cell growth were assessed by XTT-proliferation assay or dye exclusion using trypan blue and followed for up to 3 or 5 days, respectively (Fig 1B). As reported previously [13], p96^*ABL/BCR*^ alone did not induce factor-independent growth of Ba/F3 cells (Fig 1B). However, the presence of p96^*ABL/BCR*^ protein enhanced the proliferation of p185^*BCR/ABL*^-expressing cells following factor withdrawal (Fig 1B). These results were confirmed by dye exclusion assays (Fig 1B).

Factor independent growth of BCR/ABL-transduced Ba/F3 cells reflects the capacity of BCR/ABL to substitute for IL-3 survival signaling. In order to investigate the influence of p96^*ABL/BCR*^ on the transformation potential of p185^*BCR/ABL*^, we performed classical transformation assays in non-transformed Rat-1 fibroblasts. These assays consist of focus formation assays for the determination of contact inhibition and colony assays in semi-solid medium (soft agar) for the determination of anchorage-independent growth. Thus Rat-1 cells were retrovirally transduced with the constructs indicated in Fig 1A. As presented in Fig 1D, p96^*ABL/BCR*^ alone was not able to transform the Rat-1 cells; however, in combination with p185^*BCR/ABL*^, it profoundly enhanced the number of foci as compared by the Rat-1 cells expressing p185^*BCR/ABL*^ alone. The number of foci was so high that they became early confluent and were not anymore scorable at day 15, when the foci of the other samples were counted. In fact at this time point all the “carpet” of foci already was detached from the dish and fluctuating in the medium (Fig 1D).

A similar effect was seen in the colony formation in soft agar. In fact upon co-expression of p96^*ABL/BCR*^, p185^*BCR/ABL*^-positive Rat-1 cells formed a significantly higher number of colonies than with p185^*BCR/ABL*^ alone (p<0.05 or p = 0.01) (Fig 1D).

Next we sought to analyze the effects of p96^*ABL/BCR*^ and p185^*BCR/ABL*^ on the growth of human HSCs. To do this, we transduced CD34^+^/CD38^-^ PB cells obtained from G-CSF stimulated volunters with lentivirus harboring the indicated constructs (Fig 1A). Proliferation was measured at 72h after transduction. Neither empty vector nor p185^*BCR/ABL*^ alone increased the proliferation of CD34^+^/CD38^-^ cells. In contrast p96^*ABL/BCR*^ alone and the co-expression of both p96^*ABL/BCR*^ and p185^*BCR/ABL*^ increased proliferation (Fig 1E).

In order to disclose the molecular mechanism by which p96^*ABL/BCR*^ promotes the proliferative and transformative potential of p185^*BCR/ABL*^ we investigated the influence of p96^*ABL/BCR*^ on the kinase activity of p185^*BCR/ABL*^. Kinase activity was assessed by the autophosphorylation of p185^*BCR/ABL*^ as well as the p185^*BCR/ABL*^-dependent substrate phosphorylation and signaling pathway activation in Ba/F3 cells in the presence/absence of p96^*ABL/BCR*^. The autophosphorylation at Y177 in the BCR-portion as well as at Y245 in the ABL kinase domain of the p185^*BCR/ABL*^ fusion protein was strongly increased in cells co-expressing p96^*ABL/BCR*^ (Fig 1C). Expression of total p185^*BCR/ABL*^ was not affected by co-expression of p96^*ABL/BCR*^ excluding an increased p185^*BCR/ABL*^ levels as a plausible mechanism for the increased p185^*BCR/ABL*^ autophosphorylation (Fig 1C). Substrate phosphorylation was addressed on the phosphorylation status of Crkl and endogenous Bcr. Whereas Crkl phosphorylation did not increase, phosphorylation of Bcr was increased in the presence of p96^*ABL/BCR*^ (Fig 1C). BCR/ABL dependent down-stream signaling activation in the presence of p96^*ABL/BCR*^ was assessed by the phosphorylation of Stat5 and Erk1/2. The activity of BCR/ABL-dependent signaling was further enhanced by p96^*ABL/BCR*^ as evidenced by an increase of the Stat-5 activation. Also Erk 1/2 was activated as revealed by a higher amount of phosphorylated Erk 1/2 due to an up-regulation of total Erk 1/2 (Fig 1C). These findings are in accordance with both an increased phosphorylation of Bcr and an enhanced autophosphorylation in the BCR-portion of p185^*BCR/ABL*^, which are up-stream of Ras/Erk signaling.

In summary, these data show a prominent functional crosstalk between p185^*BCR/ABL*^ and p96^*ABL/BCR*^ resulting in a p96^*ABL/BCR*^-directed enhanced autophosphorylation of p185^*BCR/ABL*^ and activation of MAP-kinase pathway leading to accelerated proliferation of p185^*BCR/ABL*^-positive Ba/F3 cells and enhanced transformation potential of p185^*BCR/ABL*^.

### Targeting p96^*ABL/BCR*^ inhibits the growth of the Ph^+^ ALL cell line SupB15

**Fig 2:**
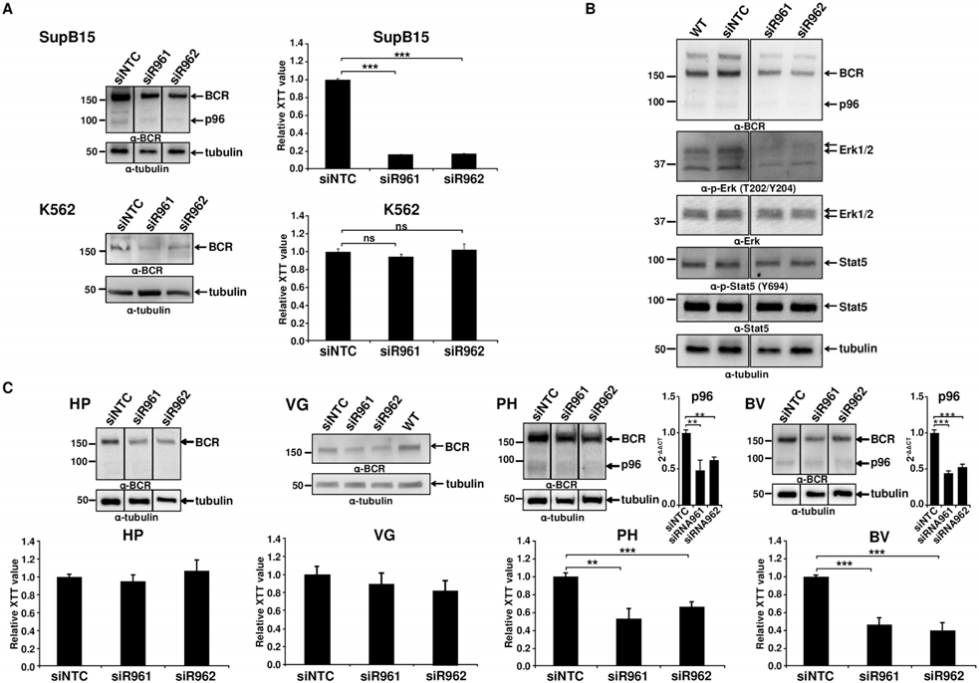
Targeting p96^*ABL/BCR*^ in Ph^+^ ALL cells. (A) SupB15 and K562 cells were lentivirally transduced with shRNA against p96^*ABL/BCR*^ (siR961 and siR962) and a control shRNA (NTC). The expression of p96^*ABL/BCR*^ and/or BCR was detected by immunoblotting using anti-BCR antibody. Tubulin was used as loading control. Proliferation was measured using XTT-assay after 3 days. One representative experiment in triplicates ± SD of at least three yielding similar results is given; (B) The effect of targeting p96^*ABL/BCR*^ by shRNA in SupB15 on STAT5 and ERK1/2 pathway was detected using the indicated antibodies; (C) Down-regulation of p96^*ABL/BCR*^ in Ph^+^ ALL PD-LTCs by shRNA. Ph^+^ ALL PD-LTCs—PH: fully TKI-responsive; BV: TKI-resistant; as controls were used: HP (Ph^-^ ALL patient) and VG: t(12;9)-TEL/ABL-positive ALL. The effect of shRNAs on the expression of p96^*ABL/BCR*^ was tested by immunoblotting using the indicated antibodies and by q-RT-PCR for PH and BV. The Ct values were normalized to that of GAPDH and results are represented as 2^-ΔΔCt^. Proliferation was measured by XTT-assay. The mean of at least experiments is given ± SD.

Based on our findings that p96^*ABL/BCR*^ exerts pro-proliferative and pro-transformative effects on p185^*BCR/ABL*^, we aimed to determine the role of p96^*ABL/BCR*^ in the growth and survival of Ph^+^ ALL cells. Thus we targeted p96^*ABL/BCR*^ in the Ph^+^ ALL cell line SupB15 cells by RNAi. The breakpoint sequence of p96^*ABL/BCR*^, the only specific sequence, was inaccessible for a rational siRNA design. Therefore we used shRNA directed against the 3'UTR of the *BCR* gene, which targeted both alleles, the der9 encoding the p96^*ABL/BCR*^ and the native *BCR* on chromosome 22. The K562 cell line was used as a control, since it harbors the M-BCR with p210^*BCR/ABL*^ and does not express the reciprocal ABL/BCR [13]. As shown by immunoblotting, lentiviral transduction of these shRNAs (efficiency of ~75%) resulted in a pronounced reduction (~80%) of p96^*ABL/BCR*^ expression as compared to non-targeting control shRNA (siNTC) whereas the expression of BCR was decreased of 40–60% (Fig 2A). No effect was seen on the expression of p185^*BCR/ABL*^ (S1A Fig). The proliferation was assessed by XTT-proliferation assay. As shown in Fig 2A, down-regulation of p96^*ABL/BCR*^ significantly reduced the proliferation rate of SupB15 cells as compared to the siNTC-transduced controls, whereas no effect was seen in K562 despite an efficient down-regulation of BCR. In order to further investigate the functional interplay between p96^*ABL/BCR*^ and p185^*BCR/ABL*^, we studied the influence of the p96^*ABL/BCR*^ knock-down on the p185^*BCR/ABL*^-dependent signaling in SupB15 cells. Down-regulation of p96^*ABL/BCR*^ almost completely abolished ERK1/2 activation, whereas no effect on the activation of STAT5 was observed (Fig 2B).

These data confirmed a functional crosstalk between p96^*ABL/BCR*^ and p185^*BCR/ABL*^ in human Ph^+^ ALL cells, which seems to be fundamental for proliferation of these cells.

### Targeting p96^*ABL/BCR*^ decreases the proliferation of PD-LTCs from Ph^+^ ALL patients and induces apoptosis

The biology of Ph^+^ ALL in adults is not fully represented by cell lines such as SupB15. Therefore, we investigated the effect of p96^*ABL/BCR*^ knock-down in different primary PD-LTCs from Ph^+^ ALL patients. We selected two different PD-LTCs: one, the PH, fully responsive to TKIs and one, BV, exhibiting a nearly complete resistance to TKIs not attributable to mutations in the TKD [22,23]. As a negative control we used a PD-LTCs, HP, from a Ph^-^ ALL patient. In order to provide additional evidence that the effects seen were due to down-regulation of p96^*ABL/BCR*^ and not to that of endogenous BCR, we utilized a TEL/ABL-expressing PD-LTC (VG) derived from a patient with t(12;9)(p13;q34)-positive ALL, previously described [24]. As the biological consequences of TEL/ABL are similar to those of BCR/ABL [25,26,27], the effects of targeted down-regulation of BCR in these cells should allow to estimate the contribution of BCR down-regulation to the effects of targeting p96^*ABL/BCR*^ in Ph^+^ ALL cells. Proliferation of the cells was measured using XTT-proliferation assay. Efficient down-regulation of p96^*ABL/BCR*^ and/or BCR in the PD-LTCs was assessed by immunoblotting and in PH and BV also by q-RT-PCR for p96^*ABL/BCR*^ (Fig 2C). The down-regulation of p96^*ABL/BCR*^ efficiently inhibited proliferation of BV (50–67%) and PH (40–45%), but had no effect on HP cells (max. 10%) and the effect on VG cells was weak (8–20%)(Fig 2C). These findings not only show that the down-regulation of p96^*ABL/BCR*^ and not that of BCR is responsible for the block of proliferation but also indicate that endogenous BCR does not play any role for the leukemogenic potential of either TEL/ABL or BCR/ABL. This confirms several studies showing that BCR has even a negative impact on BCR/ABL activation and its oncogenic potential [28,29,30,31]. In order to investigate whether apoptosis is important for the inhibition of proliferation, we stained the Ph^+^ PD-LTCs cells with 7-AAD and measured the apoptosis rate by flow cytometry. As shown in S1B Fig, both specific shRNAs induced a high rate of apoptosis (about 40% and 50%, respectively) in the Ph^+^ PD-LTCs whereas no effect (10–15%) was seen with the siNTC transduced controls (S1B Fig).

In summary our data implicate a role for p96^*ABL/BCR*^ in the survival of Ph^+^ PD-LTCs with m-BCR which harbors both p185^*BCR/ABL*^ and the reciprocal p96^*ABL/BCR*^ fusion proteins.

### Targeting p96^*ABL/BCR*^ increases responsiveness of Ph^+^ PD-LTCs to ABL-kinase inhibitors

**Fig 3:**
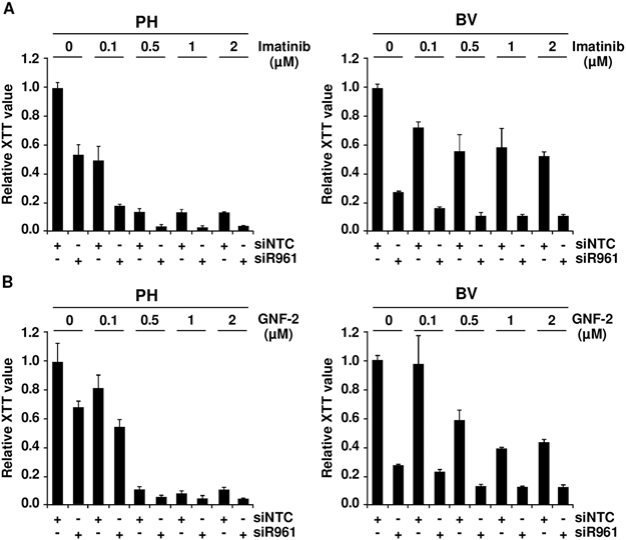
Role of p96^*ABL/BCR*^ for response to selective TKI. PH and BV cells were transduced lentivirally with shRNAs against p96^*ABL/BCR*^; (A) Treatment with different concentrations of imatinib (0.1, 0.5, 1 and 2 μM). All experiments were performed in triplicates for a total of three experiments, which gave similar results. One representative experiment is given ± SD; (B) Treatment with different concentrations of GNF-2 (0.1, 0.5, 1 and 2 μM). All experiments were performed in triplicates for a total of three, all yielding similar results. One representative experiment is given ± SD.

If the crosstalk between p96^*ABL/BCR*^ and p185^*BCR/ABL*^ leads to an increased kinase activity, in a reverse conclusion the down-regulation of p96^*ABL/BCR*^ should increase the responsiveness of Ph^+^ ALL cells towards selective ABL-kinase inhibitors. In order to test this hypothesis, we used two different classes of TKIs, imatinib, a classical ATP-competitor, and GNF-2 an allosteric inhibitor which binds to the myristoyl binding pocket of ABL [32,33]. The PH and the resistant BV PD-LTCs were exposed to increasing concentrations (0.1 to 2μM) of imatinib or GNF-2 in the absence/presence of the shRNA targeting p96^*ABL/BCR*^. Proliferation was measured by XTT-assay. As shown in Fig 3A in the presence of the NTC control shRNA, PH cells exhibited a full response to imatinib whereas the BV cells only weakly responded to imatinib even at higher dosages. The presence of the specific shRNA not only increased the response of PH cells but fully restored to the growth inhibition by imatinib in BV cells (Fig 3A). Very similar results were obtained by using the allosteric inhibitor GNF-2 (Fig 3B).

These results indicate that targeting p96^*ABL/BCR*^ in primary PD-LTCs of Ph^+^ ALL increases response to selective ABL inhibitors.

### Co-expression of p96^*ABL/BCR*^ and p185^*BCR/ABL*^ increases serial replating potential of murine fetal liver hematopoietic stem and progenitor cells (HSPCs)

**Fig 4:**
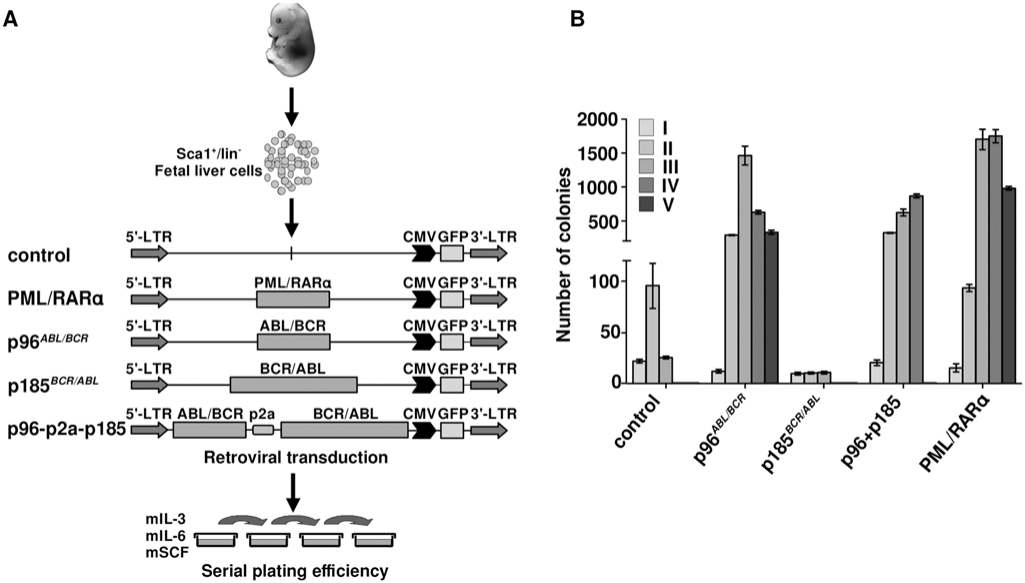
Effect of the t(9;22) fusion proteins the serial replating potential of murine fetal liver HSCs. (A) Schematic representation of the experimental procedure. Sca1^+^/lin^-^ cells were immunomagnetically isolated from murine fetal liver and the cells were transduced with the indicated retroviruses and plated in semi-solid medium supplemented with growth factors for determination of the serial replating potential; (B) Colony numbers were counted on day 10, cells were harvested and serially replated (I-VI-plating rounds).

Ph^+^ ALL has been proposed to originate from a BCR/ABL-mediated transformation at the level of committed progenitor cells. In addition, inactivation of BCR/ABL by TKIs seems to be ineffective regarding the eradication of the disease. In order to disclose a role for p96^*ABL/BCR*^ in a leukemic stem cell model, we compared the effects of p96^*ABL/BCR*^ and p185^*BCR/ABL*^ on the biological characteristics of immature HSCs in serial replatings in semi-solid medium. Therefore we isolated Sca1^+^/lin^-^ cells from murine fetal liver and transduced them retrovirally with the constructs (schematic procedure in Fig 4A). PML/RARα was used as a positive control, due to its well-known effect on the self-renewal of HSPCs [34,35,36]. The successful transduction was verified by flow cytometry (S2 Fig). As shown in Fig 4B, the replating efficiency of the empty vector and p185^*BCR/ABL*^-transduced cells was limited to three cycles of replating. In contrast, p96^*ABL/BCR*^ alone increased the number of serial replatings with an increase in the number of CFUs in each round of replating similar to PML/RARα (Fig 4B). The co-expression of p185^*BCR/ABL*^ seemed to inhibit the serial replating capacity of HSPC expressing p96^*ABL/BCR*^ alone, without suppressing it to the level of control and only p185^*BCR/ABL*^ expressing HSPC (Fig 4B).

Our observation show an autonomous role of p96^*ABL/BCR*^ in immature HSPCs, which is stronger than that of p185^*BCR/ABL*^ alone and is not suppressed by the co-expression of p185^*BCR/ABL*^.

### Co-expression of p96^*ABL/BCR*^ and p185^*BCR/ABL*^ increases HSPCs-derived CFU-S12

Our previous data suggest that ABL/BCR fusion proteins target immature HSPCs thereby influencing their commitment [13]. In order to determine the effect of both p96^*ABL/BCR*^ and p185^*BCR/ABL*^ on the self-renewal of early HSPCs and to address the question whether there is a functional hierarchy between p96^*ABL/BCR*^ and p185^*BCR/ABL*^ regarding their role in the maintenance and proliferation of subpopulations in the stem cell compartment, we performed CFU-S12 assays. The CFU-S12 performed in combination with a 9 days culture *in vitro* (in which normal control cells lose their CFU-S12 potential by spontaneous differentiation) reveals a combined effect of a given transgene on the proliferation/self-renewal and the differentiation of HSPCs. Only a differentiation block maintains the potential of the HSPCs to give origin to colonies in a CFU-S12 [37].

**Fig 5:**
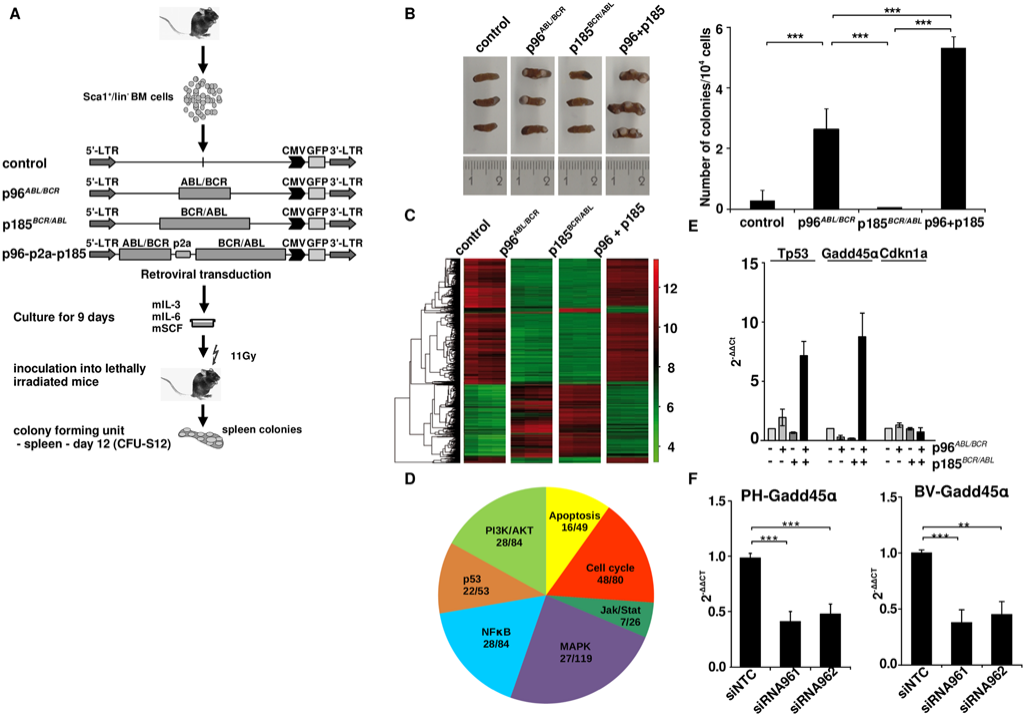
Stem cell colonogenic potential of t(9;22) fusion proteins. (A) Experimental strategy for studying the influence of t(9;22) fusion proteins on the biology of murine HSCs. Sca1^+^/lin^-^ bone marrow (BM) cells were infected with the indicated retroviruses and maintained for 9 days in liquid culture supplemented with the indicated growth factors. 1 x 10^4^ cells were inoculated into lethally irradiated recipients that were sacrificed at day 12 after transplantation; (B) Number of colonies in the spleens (n = 3); the experiment was performed a total of three times with similar results. One representative experiment is given; (C) Gene expression profile induced by t(9;22) fusion proteins in spleen from the CFU-S12. Clustering was done by selecting genes with the highest SD and sorted according to the similarity in expression level; (D) *Seven representative cellular pathways known to be influenced by BCR/ABL related to cell cycle regulation*, *proliferation and apoptosis are presented here*. *The numbers indicate the number of differentially expressed genes between p185* ^*BCR/ABL*^ *and p96* ^*ABL/BCR*^ + p185^*BCR/ABL*^ (p96+p185)-positive cells from the total number of the genes (related to each of the pathways); (E) Total RNA was isolated from spleens from the CFU-S12. The expression levels of Tp53, Gadd45α, and Cdkn1a were analyzed using q-RT-PCR. The Ct values were normalized to that of Gapdh and results are represented as 2^-ΔΔCt^. The mean of three independent experiments each done in triplicates is given ± SD; (F) PH and BV were lentivirally transduced with shRNA against p96^*ABL/BCR*^ (siR961 and siR962) and a control shRNA (NTC). The expression of GADD45α was detected by q-RT-PCR. The Ct values were normalized to that of GAPDH and results are represented as 2^-ΔΔCt^.

Therefore Sca^+^/lin^-^ HSPCs were isolated and retrovirally transduced with the transgenes (schematic procedure in Fig 5A). After 9 days in culture, cells were transplanted into lethally (11Gy) irradiated recipients. The spleens were isolated after 12 days and colonies were counted (Fig 5A). As shown in Fig 5B, p96^*ABL/BCR*^ increased number of spleen colonies as compared to empty vector controls or mice transplanted with cells expressing p185^*BCR/ABL*^. Co-expression of p96^*ABL/BCR*^ and p185^*BCR/ABL*^ resulted in additional significant increase of both number and size of colonies (Fig 5B).

Taken together, these data show a differential effect of p96^*ABL/BCR*^ as compared to p185^*BCR/ABL*^, most likely due to a differentiation block of HSPCs, which is further increased by the co-expression of both p96^*ABL/BCR*^ and p185^*BCR/ABL*^.

### Co-expression of p96^*ABL/BCR*^ and p185^*BCR/ABL*^ differentially regulates expression of cell cycle and apoptosis genes and leads to an up-regulation of Tp53 and Gadd45α but not of Cdkn1a

In order to disclose the mechanisms underlying the different effects of p96^*ABL/BCR*^ and p185^*BCR/ABL*^ alone and in combination on HSPCs, we compared the gene expression profiles induced by p96^*ABL/BCR*^, p185^*BCR/ABL*^ and the combination of both using CFU-S12 spleens by microarray analysis (S1 Text). The analysis was performed in triplicates for each construct, empty vector (control), p96^*ABL/BCR*^, p185^*BCR/ABL*^ and p96^*ABL/BCR*^-p185^*BCR/ABL*^. An unsupervised clustering grouped the profiles of the triplicates together (Fig 5C). Due to the wide range of differentially expressed genes between p185^*BCR/ABL*^ and p96^*ABL/BCR*^-p185^*BCR/ABL*^ samples, we analyzed in greater detail those signaling pathways known to be important for the pathogenesis of Ph^+^ leukemia in which at least 5 genes were differentially regulated. These pathways were related to cell cycle regulation, proliferation and apoptosis (Fig 5D). The schematic representation of genes involved in these pathways is represented in S3–S8 Figs. This analysis revealed an increase of Tp53 expression upon co-expression of p96^*ABL/BCR*^ and p185^*BCR/ABL*^. The up-regulation of Tp53 was accompanied by an up-regulation of Gadd45α, but not of Cdkn1a, both main Tp53 target genes in the DNA-damage response (Fig 5D). In order to validate the microarray data, q-RT-PCR was performed on the RNA derived from CFU-S12 and the results were in agreement with those obtained in the expression profiling (Fig 5E).

To confirm the significance of these findings for the human Ph+ ALL we studied the GADD45α expression in PD-LTCs of Ph^+^ ALL. Therefore we targeted p96^*ABL/BCR*^ in PH and BV PD-LTCs by the above described shRNA and revealed a significant reduction of GADD45α expression by q-RT-PCR (Fig 5F).

Up-regulation of Tp53 and Gadd45α is related to DNA-repair processes [38]. In order to disclose a relationship between co-expression of p96^*ABL/BCR*^ and p185^*BCR/ABL*^ and increased DNA-repair we compared the γH2AX foci in p185^*BCR/ABL*^-positive and p96^*ABL/BCR-*^p185^*BCR/ABL*^-positive leukemic spleens as a marker for DNA-repair at sites of double-strand breaks (DSB)(S1 Text)[39]. We found in p185^*BCR/ABL*^-positive spleens an increased number of signals, many of them abnormal [40] and most likely related to the high number of apoptotic granulocytes (S9 Fig). In contrast p96^*ABL/BCR-*^p185^*BCR/ABL*^-positive leukemic spleens showed a high number of cells with γH2AX foci, indicating an increased number of DSBs with ongoing DNA repair (S9 Fig). These data strongly suggest that the up-regulation of Tp53/Gadd45α is related to increased number of DSBs upon co-expression of p96^*ABL/BCR*^ in p185^*BCR/ABL*^-positive leukemia.

Given the fact that Gadd45α can be regulated also independently of Tp53 [38] we wondered whether the up-regulation of Gadd45α may contribute by itself to the biological effects of p96^*ABL/BCR*^. Therefore we lentivirally expressed Gadd45α in early primary Sca1^+^/lin^-^ HSPCs (S1 Text). We kept these cells either in liquid culture or plated them in semi-solid medium both supplemented with mIL-3, mIL-6 and mSCF. The cells in semi-solid medium were harvested, counted and replated as described above. In both conditions the expression of Gadd45α led to an increased proliferation which led to an increased colony number as compared to empty vector transduced controls in the serial plating rounds (S10 Fig).

### p185^*BCR/ABL*^ alone induces a CML-like disease whereas the co-expression with p96^*ABL/BCR*^ leads to leukemia with an ALL phenotype

**Fig 6:**
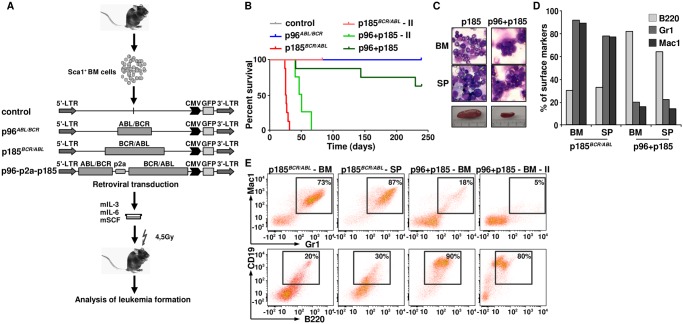
The leukemogenic potential of t(9;22) fusion proteins. (A) Schematic representation of the experimental procedure. Sca1^+^ bone marrow cells were infected with the indicated retroviruses and inoculated into sub-lethally irradiated mice. Empty vector-transduced cells were used as control; (B) Kaplan Maier curves present the probability of survival upon primary induction of leukemia and re-transplantation of leukemic cells in order to induce secondary (II) leukemia; (C) May-Grünwald-Giemsa staining of cytospins from BM and spleens of p185^*BCR/ABL*^ and p96+p185-positive leukemia of one representative mouse in each group; (D) Expression of differentiation specific surface markers (Mac1: monocytes- macrophage, Gr1: granulocytes and B220: mature B-cells) of one representative mouse in p185^*BCR/ABL*^ and *p96* ^*ABL/BCR*^ + p185^*BCR/ABL*^ (p96+p185) groups. (E) Co-expression of differentiation specific surface markers (Mac1/Gr-1—myeloid leukemia; B220/CD19: B-cell leukemia) of one representative mouse in p185^*BCR/ABL*^ and *p96* ^*ABL/BCR*^ + p185^*BCR/ABL*^ (p96+p185) groups and secondary transplanted sup.

In order to further investigate the consequences of the co-expression of p96^*ABL/BCR*^ and p185^*BCR/ABL*^ for leukemogenesis, we addressed the question of whether the presence of p96^*ABL/BCR*^ affects the phenotype or initiation of p185^*BCR/ABL*^-mediated leukemia. Therefore we transduced Sca1^+^ murine HSPCs with p96^*ABL/BCR*^, p185^*BCR/ABL*^ and the provirus encoding both proteins and inoculated the transduced HSPCs into sub-lethally (4,5 Gy) irradiated recipients. Recipients inoculated with empty vector transduced Sca1^+^ cells served as controls (Fig 6A). As already known, all the mice inoculated with p185^*BCR/ABL*^-transduced cells rapidly developed a CML-like myeloproliferative disease defined by splenomegaly (400–1200 mg spleen weight) and high numbers of Mac1 (monocytes- macrophage) and Gr1 (granulocytes) and a low number of B220 (mature B-cell) expressing cells (Fig 6C and 6D). Notably, the co-expression of p96^ABL/BCR^ and p185^ABL/BCR^ induced a lymphoid-like leukemia phenotype in 37% of the mice, with the majority of BM cells expressing the B220, and only few myeloid cells and associated with moderate splenomegaly (90–300mg spleen weight)(Fig 6B–6D). As compared to the CML-like disease induced by p185^*ABL/BCR*^ alone, the onset of the B-cell leukemia was delayed. In contrast to the CML-like disease the p96^*ABL/BCR*^ and p185^*ABL/BCR*^ induced lymphoid-like leukemia was re-transplantable, giving origin to a full blown leukemia within 38–60 days (Fig 6B). The secondary leukemia exhibited a surface marker phenotype identical to that of the primary leukemia characterized by a great majority of B220/CD19-positive blasts confirming the B-cell origin of these leukemias (Fig 6E).

In summary, these data indicate that the co-expression of p96^*ABL/BCR*^ and p185^*BCR/ABL*^ shifts the leukemia from a myeloproliferative disease upon p185^*BCR/ABL*^ alone to ALL.

## Discussion

Our understanding of the pathogenesis of Ph^+^ leukemias has improved, but several key features remain unexplained, such as the association of the m-BCR with Ph^+^ ALL and different responses of (m-BCR) p185^*BCR/ABL*^ and (M-BCR) p210^*BCR/ABL*^-positive leukemia to TKIs. In contrast to CML-CP, which is maintained by p210^*BCR/ABL*^ alone [41], many findings suggest that in Ph^+^ ALL BCR/ABL needs additional factors for the induction and maintenance of the disease.

Although the t(9;22) is a balanced translocation, one of the most obvious factors, the reciprocal ABL/BCR fusion protein was early abandoned, because no relationship between the presence of the transcripts and clinical features, like prognosis, therapy response, or others was seen [42]. These observations, in a still pre-TKI era, were exclusively based on CML, but until today nothing is known about a role for p96^*ABL/BCR*^ in Ph^+^ ALL, where the transcript is present in 100% of the cases with m-BCR and efficiently translated [13,16].

Our here presented data further confirms that the ABL/BCR contributes to the maintenance of the disease. This is supported by the fact that a siRNA-mediated targeting of ABL/BCR strongly reduced the proliferation not only of an ALL cell line, but also of primary Ph^+^ PD-LTCs, which was accompanied by the induction of apoptosis.

The cell growth arrest and apoptosis upon down-regulation of ABL/BCR strongly suggests a functional interplay between the two fusion proteins in Ph^+^ ALL. This is further confirmed by our findings that ABL/BCR increases transforming kinase activity of BCR/ABL leading to a higher proliferation rate in factor-dependent Ba/F3 cells, primary human CD34^+^/CD38^-^ HSCs, a tremendously increased colony formation in the classical transformation assays in untransformed fibroblasts and an increased CFU-S12 formation. The functional interplay between the two fusion proteins seems to be based on an enhanced kinase activity of BCR/ABL in the presence of ABL/BCR leading to an enhanced activity of Erk kinase responsible for an increased transformation potential of BCR/ABL. MAP kinase pathway activates the expression of its downstream target genes; therefore it still has to be investigated if this alters the phosphorylation of BCR/ABL upstream of this protein.

The functional interplay enhances the expression of Tp53 and one of its target genes, Gadd45α, but not that of Cdkn1a most likely as a response to an increased number of DSBs in cells co-expressing BCR/ABL and ABL/BCR leading to DNA-repair but not to apoptosis or senescence. Why the up-regulation of Tp53 activated only Gadd45α but not Cdkn1a, is still unclear. An explanation could be the capacity of Tp53 to regulate long intergenic non coding RNAs (lncRNA) which may play a role in the regulation of Cdkn1a expression [43,44]. On the other hand the consequences of an up-regulation of Tp53 in hematopoietic cells seem also to be dependent on their differentiation status. Only in committed progenitors the X-ray induced up-regulation of Tp53 leads to apoptosis, whereas in short-term hematopoietic stem cells the induction of Tp53 is not followed by apoptosis, suggesting a different DSB responses in stem cell and progenitor populations with a different differentiation potential [45]. Given the fact that Cdkn1a is a an attenuator of BCR/ABL-mediated cell proliferation [46], its down-regulation in the presence of ABL/BCR together with activation of Gadd45α may contribute to the enhanced transformation potential of BCR/ABL. To which extent the Gadd45α activation is involved in the pathogenesis of Ph+ ALL remains to be disclosed. On the other hand Gadd45α does not exhibit growth suppressing functions, as suggested by our findings that Gadd45α is able to increase proliferation of murine HSPCs. This is in accordance to findings showing that up-regulation of GADD45 does not only protect hematopoietic progenitors from UV-induced apoptosis by the activation of p38-NFκB signaling [47,48], but it represents together with an activation of ERK-1 a negative prognostic factor in malignant lymphoproliferative diseases [49]. Our data indicate that Ph^+^ ALL may be one of the malignant diseases in which Gadd45α exhibits a pro-oncogenic function [50].

The functional interplay between BCR/ABL and ABL/BCR may promote the disease in the absence of BCR/ABL inhibition, whereas the functional independence of ABL/BCR as a self-standing leukemogenic factor may contribute to the maintenance of the disease upon an efficient BCR/ABL inhibition and thus to the only transient response of Ph^+^ ALL patients to the TKI treatment. The functional independence of ABL/BCR is given not only by the increased serial replating efficiency and CFU-S12, as compared to BCR/ABL and controls, but also by its capacity to induce a leukemic phenotype in syngenic mice [13]. One could hypothesize that there may exist a functional hierarchy with BCR/ABL active in committed progenitors and ABL/BCR active at an earlier stage of differentiation, which contributes to the maintenance of the leukemia even upon an efficient BCR/ABL inhibition and the following selection of subclones with BCR/ABL harboring resistance mutations.

A functional hierarchy between the t(9;22) fusion proteins could play an important role for the fate decision of leukemia. From the Sca1^+^ compartment BCR/ABL is able to induce nearly exclusively myeloproliferative disease, which is characterized by a rapid onset of the disease, accumulation of mature myeloid linage cells in the spleen and BM and splenomegaly. However an ALL-like phenotype appeared when we co-expressed both p96^*ABL/BCR*^ and p185^*BCR/ABL*^ even with a lower incidence and a much longer latency as compared to BCR/ABL alone. The shift from myeloid to lymphatic phenotype may be due to the increased BCR/ABL kinase activity in the presence of p96^*ABL/BCR*^ as it has been already shown by Jones and co-worker for the development of myeloid or lymphatic BC in patients with CML [10]. The fact that not all of these mice develop the disease can be attributed most likely to the fact that the target cells for leukemic transformation followed by an acute leukemia phenotype by p96^*ABL/BCR*^ and p185^*BCR/ABL*^ are rarer, as compared to that targeted by BCR/ABL for induction of myeloproliferation. In addition, a different grade of proliferative advantage between the two scenarios may account for the differences in latency.

Taken together, our study provide clear evidence of a functional interplay between BCR/ABL and ABL/BCR in the pathogenesis of Ph^+^ ALL, which suggest an additional target to be hidden by molecular therapy approaches in order to achieve an efficient treatment of this high risk subgroup of ALL.

## Materials and Methods

### Plasmids

The cDNAs encoding p185^*BCR/ABL*^ PML/RARα and p96^*ABL/BCR*^ were described previously [17,51,52]. For the simultaneous expression of genes, p2a peptide-linked multi-cistronic retroviral vector was used, which allows the expression of multiple proteins from a single open reading frame (ORF)[53]. The p2a sequence was kindly provided by Frank Schnütgen (University Clinic, Frankfurt, Germany). In order to construct pEp96^*ABL/BCR*^-p2a-p185^*BCR/ABL*^ the p2a fragment was amplified by PCR using Pr1, 5′- gcg gcc gcg agc cac gaa ctt ctc-3′and Pr2, 5′- ggt cag taa att gga tat cgg ccc-3′ and transferred via TA cloning into the pCR2.1 vector (Invitrogen, Karlsruhe, Germany) and controlled by Sanger sequencing. Then it was transferred by EcoRV/NotI into EcoRV/NotI digested pEp96^*ABL/BCR*^. As next step for a continuous ORF, stop codon of the p96^*ABL/BCR*^ sequence was deleted using the “quick change site-directed mutagenesis” kit (Stratagene, La Jolla, CA, USA) using Pr3, 5′-ttc tcc acc gaa gtc aag aat tcg cgg ccg-3′ and Pr4, 5′-cgg ccg cga att ctt gac ttc ggt gga gaa-3′. FseI and SacII sites were introduced in p96^*ABL/BCR*^-p2a fragment at the 5' and 3', respectively, by PCR using the following primers: Pr5, 5′-acc cgc gga tgt tgg aga tct gc-3′ and Pr6, 5′-ggc cgg cct tcg gcc cgg ggt ttt-3′. The resulting construct was then subcloned into the FseI/SacII-digested pEp185^*BCR/ABL*^. All PCR-products were controlled by Sanger sequencing. Thus the final construct was available in the Gateway^®^ entry-vector (pENTR1A) for recombination into destination Gateway^®^ vectors according to the manufacturer's instructions (Invitrogen). All retroviral expression vectors used in this study were based on PINCO as previously described [54]. Lentiviral vectors expressing short hairpins against human ABL/BCR and non-targeting control lentiviral vectors were based on the PLKO-1 vector (Sigma, Steinheim, Germany). The shRNAs were designed as siR961: ccg gca gat cca gat acc taa gct cga gct tat tag gta tct gga tct gtt ttt tg, and siR962: ccg gca aga gtt aca cgt tcc tga tct cga gat cag gaa cgt gta act ctt gtt ttt.

### Cell lines, inhibitors

Ecotropic Phoenix, 293T packaging cells, and Rat-1 fibroblasts were cultured in Dulbecco’s modified Eagle medium (DMEM; sigma) supplemented with 10% FCS (Invitrogen, Karlsruhe, Germany). K562 and SupB15 cells were kept in RPMI 1640 containing 10% or 15% FCS, respectively. Ba/F3 cells were grown in RPMI + 10% FCS supplemented with 10ng/mL mIL-3 (Cell Concepts, Umkirch, Germany). Ph^+^ ALL patient-derived long-term cultures (PD-LTCs)(PH, BV, VG and HP) were maintained in a serum-free medium as described previously [24,55]. Imatinib (kindly provided by Novartis, Basel, Switzerland) and GNF-2 (Sigma) were dissolved in DMSO for a stock solution and diluted to the appropriate concentrations.

### Isolation of Sca1^+^ and Sca1^+^/lin^-^ HSCs

All animal studies were performed in accordance with international animal protection guidelines and approved by the Regierungspräsidium Darmstadt (approval number F39/08). Sca1^+^ and Sca1^+^/lin^-^ HSCs were isolated from 8 to 12 week-old female C57BL/6J mice (Janvier, St. Berthevin, France) as described. The cells were ‘‘lineage depleted” by labeling the cells with biotin-conjugated lineage panel antibodies against B220, CD3e, Gr1, Mac1 and Ter-119 (Miltenyi, Bergisch-Gladbach, Germany). Labeled cells were removed using ‘‘MACS” cell separation columns according to the manufacturer's instructions. Sca1^+^ cells were purified by immunomagnetic beads using the ‘‘MACS” cell separation columns according to the manufacturer’s instructions (Miltenyi). Prior to further use, the purified cells were pre-stimulated in medium containing mIL-3 (20 ng/mL), mIL-6 (20 ng/mL) and mSCF (100 ng/mL)(Cell Concepts).

### Enrichment of CD34^+^/CD38^-^ cells

The source of CD34^+^/CD38^-^ was residual peripheral blood (PB) of healthy donors stimulated with G-CSF for the mobilization for stem cell transplantation kindly provided by Halvard Bönig (German Red Cross Blood Donor Centre, Institute of Transfusion Medicine and Immunohematology, Goethe University, Frankfurt, Germany) after informed consent. CD34^+^ cells were isolated using the CD34-Multisort Kit (Miltenyi) followed by the CD38 depletion by labeling the cells with FITC-conjugated anti CD38 antibody which allowed the immunomagnetic isolation by a MACS separation column according to the manufacturer's instructions (Miltenyi).

### Retro- and lentiviral infection

Retro- and lentiviral supernatant using ecotropic Phoenix and 293T packaging cell lines were obtained as described [56]. 24-well plates were first coated with retronectin (Takara Bio Inc., Otsu, Japan) followed by the retro- or lentiviral supernatant. Then target cells (10^5^ cells/mL) were plated and incubated overnight by 37°C. Subsequently another two rounds of infection were performed by adding viral supernatant and centrifuged at 2.200 rpm for 45 minutes by 32°C. Infection efficiency was measured after 48h by determining the percentage of GFP-positive cells by fluorescence-activated cell sorting (FACS). Differences in the infection efficiency between samples did not exceed 10%.

### Cell growth, proliferation and apoptosis

Proliferation was assessed by using XTT proliferation kit (Roche, Mannheim, Germany), according to the manufacturer’s instructions. Cell growth was assessed by dye exclusion using Trypan-blue according to widely used protocols. Apoptosis was measured by the 7-amino-actinomycin D (7-AAD) method as described before [52,57].

### Transformation assay

After retroviral transduction 5 x 10^3^ transduced Rat-1 cells were suspended in ‘top-agar’, DMEM supplemented with 10% FCS and 0.25% bacto-agar (DIFCO Laboratories, Detroit, USA), and stacked in six-well plates filled with DMEM supplemented with 10% FCS and 0.5% bacto-agar (2 ml per well). After 15 days incubation at 37°C and 5% CO_2_ colonies were counted. The focus-formation assays were performed in 24-well plates. 4 x 10^4^ transduced Rat-1 cells/well were plated. Unstained foci were photographed at day 15 using an AxioCam HRc system (Zeiss, Goettingen, Germany) with 10x magnification.

### Immunoblotting

Immunoblot analyses were performed according to widely established protocols using the following antibodies: anti-ABL (α-ABL), anti-BCR (α-BCR) (St. Cruz Biotechnology, Santa Cruz, USA), anti- phosphorylated ABL (α-p-ABL-Y245), anti-CRKL (α-CRKL), and anti-phosphorylated CRKL (α-p CRKL-Y207), anti-STAT5 (α-STAT5), and anti-phosphorylated STAT5 (α-p STAT5-Y694), anti-ERK (α-ERK), and anti-phosphorylated ERK (α-p ERK-T202-Y204) and anti-phosphorylated BCR (α-p BCR-Y177) (Cell Signaling, Boston, USA), and anti-Tubulin (α-Tubulin)(Neo Markers, Asbach, Germany). Membrane blocking and antibody incubation were performed in 5% low-fat dry milk, followed by washing in Tris-buffered saline (TBS) (10 mM Tris-HCl pH 8, 150 mM NaCl) containing 0.1% Tween20 (TBS-T). The membrane was then incubated with the secondary horse-radish-peroxidase-conjugated antibody. After extensive washing with TBS-T Signal was detected by chemiluminescence using the ECL detection system (Thermo Scientific, Schwerte, Germany). Blots were “stripped” using Restore Western blot Stripping Buffer Pierce (Perbio Science, Bonn, Germany). The quantification of the protein bands was performed with Image Studio 4.0 Imaging Software (LI-COR Biosciences, Lincoln, NB, USA).

### Colony forming unit (CFU) assays and serial replating

At day 2 post-infection, Sca1^+^/lin^-^ cells were plated at 5 x 10^3^ cells/mL in methyl-cellulose supplemented with mIL-3 (20 ng/mL), mIL-6 (20 ng/mL) and mSCF (100 ng/mL)(Stem-Cell Technologies, Vancouver, Canada). On day 10 after plating, the number of colony forming units (CFUs) was determined. After washing out from the methyl-cellulose, the cells were stained with specific antibodies for the detection of surface marker expression by FACS. 5 x 10^3^ cells/ml were replated in methyl-cellulose, thus permitting determination of the serial replating potential.

### Day 12 spleen colony-forming unit (CFU-S12) assay

Sca1^+^/lin^-^ cells were retrovirally transduced and plated in 24 well in the presence of mIL-3, mIL-6 and mSCF. After 9 days of culture 1 x 10^4^ infected cells were inoculated intravenously into lethally (11Gy) irradiated recipient mice. At day 12 spleens of 3/6 mice/group were fixed in Tellesnizky’s fixative for counting the colonies and 3 were used for RNA isolation and subsequent gene expression and RT-PCR analysis.

### Syngeneic transduction/transplantation model of leukemia, flow cytometry

Female C57BL/6J mice 8–12 weeks of age (Janvier) were used as recipients and donors. 1x10^5^ transduced Sca1^+^ cells were inoculated into sub-lethally irradiated (4.5 Gy) recipient mice via tail vein injection. The mice were sacrificed at the first appearance of morbidity (weight loss >10%, neurological abnormalities, failure to thrive or diarrhea). Statistical relevance was determined by the Log-rank test. Cytospins of whole bone marrow and spleen cells were stained with May-Grünwald-Giemsa stain. For secondary transplantation the frozen spleen cells from the primary leukemic mice were thawed and 10^4^ cells/mouse were inoculated into sublethally (4.5Gy) irradiated recipients. For surface marker analysis freshly thawed cells (5x10^5^/sample) were stained with B220 (V450), CD19 (PE), Gr1 (PerCP-Cy5.5) and Mac1 (APC) according to manufacturer's instructions (Beckton Dickinson Biosciences, Heidelberg, Germany). Analysis were performed on a FACS Canto II (Beckton Dickinson).

### Gene expression array

RNA was isolated from CFU-S12 spleens using RNeasy kit according to the manufactures protocol (Qiagen, Düsseldorf, Germany). The cDNA synthesis was performed using standardized protocols (Applause WT-Amp Plus ST Systems and Encore Biotin Module, NuGEN (Bemmel, Niederlands). Microarray hybridization to GeneChip MoGene 1.0-ST-V1 (Affymetrix, Santa Clara, CA, USA), washing steps and scanning of the microarray were performed according to Affymetrix protocol. Heatmaps were done with the Spotfire software (Spotfire Decision Site 9.1.2, TIBCO Spotfire, Boston, MA, USA). The statistical analysis was done with the statistical computing environment R version 2.12 [58]. Additional software packages were taken from the Bioconductor project [59].

### Real time PCR

Total RNA and first strand cDNA were obtained from CFU-S12 spleens as described above. The TaqMan PCR was conducted in triplicates in total of two times following standard protocols using the ABI PRISM 7700 (Applied Biosystems, Darmstadt, Germany). For the quantification of Tp53, Gadd45α, and Cdkn1a mRNA, gene expression quantification using ‘‘Assay-on-demand” was performed according to the manufacturer's instructions (Applied Biosystems, Foster City, CA, USA). For the detection of ABL/BCR-transcripts on PH and BV cells the following primers probes were used: AB-a-fw: 5'-cct cgt cct cca gct gtt a-3'; AB-a rev: 5'-gcc gta tcc agg tgg tgt-3'; AB-a probe: 5'Fam—tcc gaa cga gcc atc ttc cag a—3'Tamra; AB-b-fw: 5'-gaa tca tcg agg cat ggg-3'; AB-b-rev; 5'-ccg tat cca ggt gtt c-3'; AB-b probe 5'Fam—cga acg agc cat gtt cca ca-3'Tamra. Normalization to glyceraldehyde-3-phosphate dehydrogenase (Gapdh) was done for each sample. Ct values were exported into a Microsoft Excel worksheet for calculation of fold changes according to the comparative Ct method. The amount of target, normalized to endogenous Gapdh is given by 2^-ΔΔCt^ [60].

### Statistical analysis

All statistical analyses were performed using Student’s-*t*-test and p ≤ 0.05 was considered as significant. All experiments were performed at least 3 times and the results were taken only if the replicates of independent experiments indicated the same results. GraphPad Prism 5.0 was used to provide the statistical calculations.

### Ethics statement

All animal studies were performed in accordance with international animal protection guidelines and approved by the Regierungspräsidium Darmstadt (approval number F39/08). Human cells were used in agreement with the Declaration of Helsinki with the approval of the local ethic committee (approval number 329–10).

## Supporting Information

S1 Fig Targeting p96^*ABL/BCR*^ in Ph^+^ ALL cells. (A) SupB15 cells were lentivirally transduced with shRNAs (siR961 and siR962 and siNTC as control) against p96^*ABL/BCR*^. The effect on the expression of ABL/BCR and BCR/ABL, respectively, was revealed by using the indicated antibodies; anti-tubulin staining was used for loading control. (B) Induction of apoptosis. BV and PH cells were lentivirally transduced with shRNAs (siR961 and siR962 and siNTC as control) against p96^*ABL/BCR*^ and apoptosis rate was measured using 7-AAD by FACS. One representative out of three experiments each performed in triplicates with similar results is given ± SD.
(PDF)

S2 Fig Effect of the t(9;22) fusion proteins on the biology of murine fetal liver HSCs. The expression of the transgenes used in this experiment was detected by FACS for the expression of GFP. The wild-type (WT) cells were taken as negative control for GFP expression.
(PDF)

S3 Fig Schematic representation of genes related to cell cycle regulation. The cell cycle regulation genes and their interaction upon expression of the transgenes is visualized in this picture. The colored (green and red) genes are differentially regulated between p185^*BCR/ABL*^ versus p96+p185 groups. The green colored genes are down-regulated (fold changes < -1) in p185^*BCR/ABL*^-containing spleens in comparison to spleens positive for both p96^*ABL/BCR*^ and p185^*BCR/ABL*^. The red colored genes are up-regulated (fold changes > +2) in p185^*BCR/ABL*^ in comparison to p96+p185 group.
(PDF)

S4 Fig Schematic representation of genes related to apoptosis. The genes related to apoptosis signaling and their interaction upon expression of the transgenes is visualized in this picture. The colored (green and red) genes are differentially regulated between p185^*BCR/ABL*^ versus p96+p185 groups. The green colored genes are down-regulated (fold changes < -1) in p185^*BCR/ABL*^-containing spleens in comparison to spleens positive for both p96^*ABL/BCR*^ and p185^*BCR/ABL*^. The red colored genes are up-regulated (fold changes > +2) in p185^*BCR/ABL*^ in comparison to p96+p185 group.
(PDF)

S5 Fig Schematic representation of genes related to p53 pathway. The genes related to p53 pathway and their interaction upon expression of the transgenes is visualized in this picture. The colored (green and red) genes are differentially regulated between p185^*BCR/ABL*^ versus p96+p185 groups. The green colored genes are down-regulated (fold changes < -1) in p185^*BCR/ABL*^-containing spleens in comparison to the spleen which contained both p96^*ABL/BCR*^ and p185^*BCR/ABL*^. The red colored genes are up-regulated (fold changes > +2) in p185^*BCR/ABL*^ in comparison to p96+p185 group.
(PDF)

S6 Fig Schematic representation of genes related to PI3K/AKT pathway. The genes related to PI3K/AKT pathway and their interaction upon expression of the transgenes is visualized in this picture. The colored (green and red) genes are differentially regulated between p185^*BCR/ABL*^ versus p96+p185 group. The green colored genes are down-regulated (fold changes < -1) in p185^*BCR/ABL*^- containing spleens in comparison to the spleens which contained both p96^*ABL/BCR*^ and p185^*BCR/ABL*^. The red colored genes are up-regulated (fold changes > +2) in p185^*BCR/ABL*^ in comparison to p96+p185 group.
(PDF)

S7 Fig Schematic representation of genes related to MAP-kinase pathway. The genes related to MAP-kinase signaling and their interaction upon expression of the transgenes is visualized in this picture. The colored (green and red) genes are differentially regulated between p185^*BCR/ABL*^ versus p96+p185 groups. The green colored genes are down-regulated (fold changes < -1) in p185^*BCR/ABL*^-containing spleens in comparison to the spleens positive for both p96^*ABL/BCR*^ and p185^*BCR/ABL*^. The red colored genes are up-regulated (fold changes > +2) in p185^*BCR/ABL*^ in comparison to p96+p185 group.
(PDF)

S8 Fig Schematic representation of genes related to JAK-STAT signaling pathway. The genes related to JAK-STAT signaling and their interaction upon expression of the transgenes is visualized in this picture. The colored (green and red) genes are differentially regulated between p185^*BCR/ABL*^ versus p96+p185 groups. The green colored genes are down-regulated (fold changes < -1) in p185^*BCR/ABL*^-containing spleens in comparison to the spleens positive for both p96^*ABL/BCR*^ and p185^*BCR/ABL*^. The red colored genes are up-regulated (fold changes > +2) in p185^*BCR/ABL*^ in comparison to p96+p185 group.
(PDF)

S9 Fig Confocal laser scan analysis of γH2AX staining in spleens of p185^*BCR/ABL*^- and p96^ABL/BCR-^p185^BCR/ABL^-positive leukemia. Green fluorochrome—γH2AX; phaco—phase contrast. Control—empty vector transduced control spleen. p185—p185^*BCR/ABL*^- positive leukemia; p185+p96—p96^ABL/BCR-^p185^BCR/ABL^-positive leukemia.
(PDF)

S10 Fig Effect of Gadd45α on Sca1^+^/lin^-^ HSCPs. Sca1^+^/lin^-^ cells were immunomagnetically isolated from murine BM and the cells were transduced with empty virus or with Gadd45α plated either in liquid culture or in semi-solid medium supplemented with growth factors for determination of the proliferation and serial replating potential, respectively. Proliferation was assessed by XTT at day 5 after plating. Colony numbers were counted on day 10, cells were harvested and serially replated. Each time cells were counted for the determination of cell growth (I-IV-plating rounds).
(PDF)

S1 Text Supplementary information about the microarray pre-processing, supplementary Materials and Methods, and supplementary references. (DOCX)

## References

[1] Faderl S, Talpaz M, Estrov Z, O'Brien S, Kurzrock R, et al (1999) The biology of chronic myeloid leukemia. N Engl J Med 341: 164–172. 10403855 10.1056/NEJM199907153410306

[2] Nacheva EP, Grace CD, Brazma D, Gancheva K, Howard-Reeves J, et al (2013) Does BCR/ABL1 positive acute myeloid leukaemia exist? Br J Haematol 161: 541–550. 10.1111/bjh.12301 23521501

[3] Soupir CP, Vergilio JA, Dal Cin P, Muzikansky A, Kantarjian H, et al (2007) Philadelphia chromosome-positive acute myeloid leukemia: a rare aggressive leukemia with clinicopathologic features distinct from chronic myeloid leukemia in myeloid blast crisis. Am J Clin Pathol 127: 642–650. 17369142 10.1309/B4NVER1AJJ84CTUU

[4] Barnes DJ, Melo JV (2002) Cytogenetic and molecular genetic aspects of chronic myeloid leukaemia. Acta Haematol 108: 180–202. 12432215 10.1159/000065655

[5] Cimino G, Pane F, Elia L, Finolezzi E, Fazi P, et al (2006) The role of BCR/ABL isoforms in the presentation and outcome of patients with Philadelphia-positive acute lymphoblastic leukemia: a seven-year update of the GIMEMA 0496 trial. Haematologica 91: 377–380. 16531262

[6] Foa R (2011) Acute lymphoblastic leukemia: age and biology. Pediatr Rep 3 Suppl 2: e2 10.4081/pr.2011.s2.e2 22053278 PMC3206534

[7] Vignetti M, Fazi P, Cimino G, Martinelli G, Di Raimondo F, et al (2007) Imatinib plus steroids induces complete remissions and prolonged survival in elderly Philadelphia chromosome-positive patients with acute lymphoblastic leukemia without additional chemotherapy: results of the Gruppo Italiano Malattie Ematologiche dell'Adulto (GIMEMA) LAL0201-B protocol. Blood 109: 3676–3678. 17213285 10.1182/blood-2006-10-052746

[8] Melo JV, Barnes DJ (2007) Chronic myeloid leukaemia as a model of disease evolution in human cancer. Nat Rev Cancer 7: 441–453. 17522713 10.1038/nrc2147

[9] Radich JP (2001) Philadelphia chromosome-positive acute lymphocytic leukemia. Hematol Oncol Clin North Am 15: 21–36. 11258387 10.1016/s0889-8588(05)70198-2

[10] Jones D, Luthra R, Cortes J, Thomas D, O'Brien S, et al (2008) BCR-ABL fusion transcript types and levels and their interaction with secondary genetic changes in determining the phenotype of Philadelphia chromosome-positive leukemias. Blood 112: 5190–5192. 10.1182/blood-2008-04-148791 18809762 PMC2597614

[11] Hantschel O, Rix U, Superti-Furga G (2008) Target spectrum of the BCR-ABL inhibitors imatinib, nilotinib and dasatinib. Leuk Lymphoma 49: 615–619. 10.1080/10428190801896103 18398720

[12] Barnes DJ, Melo JV (2006) Primitive, quiescent and difficult to kill: the role of non-proliferating stem cells in chronic myeloid leukemia. Cell Cycle 5: 2862–2866. 17172863 10.4161/cc.5.24.3573

[13] Zheng X, Oancea C, Henschler R, Moore MA, Ruthardt M (2009) Reciprocal t(9;22) ABL/BCR fusion proteins: leukemogenic potential and effects on B cell commitment. PLoS One 4: e7661 10.1371/journal.pone.0007661 19876398 PMC2764858

[14] Jaso J, Thomas DA, Cunningham K, Jorgensen JL, Kantarjian HM, et al (2011) Prognostic significance of immunophenotypic and karyotypic features of Philadelphia positive B-lymphoblastic leukemia in the era of tyrosine kinase inhibitors. Cancer 117: 4009–4017. 10.1002/cncr.25978 21365622 PMC5548124

[15] Melo JV, Gordon DE, Cross NC, Goldman JM (1993) The ABL-BCR fusion gene is expressed in chronic myeloid leukemia. Blood 81: 158–165. 8417787

[16] Melo JV, Gordon DE, Tuszynski A, Dhut S, Young BD, et al (1993) Expression of the ABL-BCR fusion gene in Philadelphia-positive acute lymphoblastic leukemia. Blood 81: 2488–2491. 8490164

[17] Zheng X, Guller S, Beissert T, Puccetti E, Ruthardt M (2006) BCR and its mutants, the reciprocal t(9;22)-associated ABL/BCR fusion proteins, differentially regulate the cytoskeleton and cell motility. BMC Cancer 6: 262 17090304 10.1186/1471-2407-6-262 PMC1637115

[18] Radziwill G, Erdmann RA, Margelisch U, Moelling K (2003) The Bcr kinase downregulates Ras signaling by phosphorylating AF-6 and binding to its PDZ domain. Mol Cell Biol 23: 4663–4672. 12808105 10.1128/MCB.23.13.4663-4672.2003 PMC164848

[19] Ress A, Moelling K (2006) Bcr interferes with beta-catenin-Tcf1 interaction. FEBS Lett 580: 1227–1230. 16442529 10.1016/j.febslet.2006.01.034

[20] Van Aelst L, D'Souza-Schorey C (1997) Rho GTPases and signaling networks. Genes Dev 11: 2295–2322. 9308960 10.1101/gad.11.18.2295

[21] Thomas EK, Cancelas JA, Zheng Y, Williams DA (2008) Rac GTPases as key regulators of p210-BCR-ABL-dependent leukemogenesis. Leukemia 22: 898–904. 10.1038/leu.2008.71 18354486 PMC4464749

[22] Mian AA, Metodieva A, Badura S, Khateb M, Ruimi N, et al (2012) Allosteric inhibition enhances the efficacy of ABL kinase inhibitors to target unmutated BCR-ABL and BCR-ABL-T315I. BMC Cancer 12: 411 10.1186/1471-2407-12-411 22985168 PMC3488316

[23] Badura S, Tesanovic T, Pfeifer H, Wystub S, Nijmeijer BA, et al (2013) Differential effects of selective inhibitors targeting the PI3K/AKT/mTOR pathway in acute lymphoblastic leukemia. PLoS One 8: e80070 10.1371/journal.pone.0080070 24244612 PMC3828226

[24] Nijmeijer BA, Szuhai K, Goselink HM, van Schie ML, van der Burg M, et al (2009) Long-term culture of primary human lymphoblastic leukemia cells in the absence of serum or hematopoietic growth factors. Exp Hematol 37: 376–385. 10.1016/j.exphem.2008.11.002 19135770

[25] Malinge S, Monni R, Bernard O, Penard-Lacronique V (2006) Activation of the NF-kappaB pathway by the leukemogenic TEL-Jak2 and TEL-Abl fusion proteins leads to the accumulation of antiapoptotic IAP proteins and involves IKKalpha. Oncogene 25: 3589–3597. 16434962 10.1038/sj.onc.1209390

[26] Pecquet C, Nyga R, Penard-Lacronique V, Smithgall TE, Murakami H, et al (2007) The Src tyrosine kinase Hck is required for Tel-Abl- but not for Tel-Jak2-induced cell transformation. Oncogene 26: 1577–1585. 16953222 10.1038/sj.onc.1209949

[27] Okuda K, Golub TR, Gilliland DG, Griffin JD (1996) p210BCR/ABL, p190BCR/ABL, and TEL/ABL activate similar signal transduction pathways in hematopoietic cell lines. Oncogene 13: 1147–1152. 8808688

[28] Lin F, Monaco G, Sun T, Liu J, Lin H, et al (2001) BCR gene expression blocks Bcr-Abl induced pathogenicity in a mouse model. Oncogene 20: 1873–1881. 11313935 10.1038/sj.onc.1204409

[29] Liu J, Wu Y, Arlinghaus RB (1996) Sequences within the first exon of BCR inhibit the activated tyrosine kinases of c-Abl and the Bcr-Abl oncoprotein. Cancer Res 56: 5120–5124. 8912843

[30] Liu J, Wu Y, Ma GZ, Lu D, Haataja L, et al (1996) Inhibition of Bcr serine kinase by tyrosine phosphorylation. Mol Cell Biol 16: 998–1005. 8622703 10.1128/mcb.16.3.998 PMC231082

[31] Perazzona B, Lin H, Sun T, Wang Y, Arlinghaus R (2008) Kinase domain mutants of Bcr enhance Bcr-Abl oncogenic effects. Oncogene 27: 2208–2214. 17934518 10.1038/sj.onc.1210851 PMC2585769

[32] Adrian FJ, Ding Q, Sim T, Velentza A, Sloan C, et al (2006) Allosteric inhibitors of Bcr-abl-dependent cell proliferation. Nat Chem Biol 2: 95–102. 16415863 10.1038/nchembio760

[33] Mian AA, Oancea C, Zhao Z, Ottmann OG, Ruthardt M (2009) Oligomerization inhibition, combined with allosteric inhibition, abrogates the transformation potential of T315I-positive BCR/ABL. Leukemia 23: 2242–2247. 10.1038/leu.2009.194 19798092

[34] Muller-Tidow C, Steffen B, Cauvet T, Tickenbrock L, Ji P, et al (2004) Translocation products in acute myeloid leukemia activate the Wnt signaling pathway in hematopoietic cells. Mol Cell Biol 24: 2890–2904. 15024077 10.1128/MCB.24.7.2890-2904.2004 PMC371102

[35] Wang Y, Krivtsov AV, Sinha AU, North TE, Goessling W, et al (2010) The Wnt/beta-catenin pathway is required for the development of leukemia stem cells in AML. Science 327: 1650–1653. 10.1126/science.1186624 20339075 PMC3084586

[36] Zheng X, Beissert T, Kukoc-Zivojnov N, Puccetti E, Altschmied J, et al (2004) Gamma-catenin contributes to leukemogenesis induced by AML-associated translocation products by increasing the self-renewal of very primitive progenitor cells. Blood 103: 3535–3543. 14739224 10.1182/blood-2003-09-3335

[37] Oancea C, Ruster B, Henschler R, Puccetti E, Ruthardt M (2010) The t(6;9) associated DEK/CAN fusion protein targets a population of long-term repopulating hematopoietic stem cells for leukemogenic transformation. Leukemia 24: 1910–1919. 10.1038/leu.2010.180 20827285

[38] Riley T, Sontag E, Chen P, Levine A (2008) Transcriptional control of human p53-regulated genes. Nat Rev Mol Cell Biol 9: 402–412. 10.1038/nrm2395 18431400

[39] Price BD, D'Andrea AD Chromatin remodeling at DNA double-strand breaks. Cell 152: 1344–1354. 10.1016/j.cell.2013.02.011 23498941 PMC3670600

[40] Akbay EA, Contreras CM, Perera SA, Sullivan JP, Broaddus RR, et al (2008) Differential roles of telomere attrition in type I and II endometrial carcinogenesis. Am J Pathol 173: 536–544. 10.2353/ajpath.2008.071179 18599611 PMC2475790

[41] Michor F, Iwasa Y, Nowak MA (2006) The age incidence of chronic myeloid leukemia can be explained by a one-mutation model. Proc Natl Acad Sci U S A 103: 14931–14934. 17001000 10.1073/pnas.0607006103 PMC1595453

[42] Melo JV, Hochhaus A, Yan XH, Goldman JM (1996) Lack of correlation between ABL-BCR expression and response to interferon-alpha in chronic myeloid leukaemia. Br J Haematol 92: 684–686. 8616036 10.1046/j.1365-2141.1996.00350.x

[43] Barsotti AM, Prives C (2010) Noncoding RNAs: the missing "linc" in p53-mediated repression. Cell 142: 358–360. 10.1016/j.cell.2010.07.029 20691894

[44] Zhang A, Xu M, Mo YY (2014) Role of the lncRNA-p53 regulatory network in cancer. J Mol Cell Biol. 10.1093/jmcb/mju013 PMC4034727 24721780

[45] Insinga A, Cicalese A, Faretta M, Gallo B, Albano L, et al (2013) DNA damage in stem cells activates p21, inhibits p53, and induces symmetric self-renewing divisions. Proc Natl Acad Sci U S A 110: 3931–3936. 10.1073/pnas.1213394110 23417300 PMC3593901

[46] Forster K, Obermeier A, Mitina O, Simon N, Warmuth M, et al (2008) Role of p21(WAF1/CIP1) as an attenuator of both proliferative and drug-induced apoptotic signals in BCR-ABL-transformed hematopoietic cells. Ann Hematol 87: 183–193. 17960378 10.1007/s00277-007-0400-9

[47] Hoffman B, Liebermann DA (2009) Gadd45 modulation of intrinsic and extrinsic stress responses in myeloid cells. J Cell Physiol 218: 26–31. 10.1002/jcp.21582 18780287

[48] Liebermann DA, Hoffman B (2007) Gadd45 in the response of hematopoietic cells to genotoxic stress. Blood Cells Mol Dis 39: 329–335. 17659913 10.1016/j.bcmd.2007.06.006 PMC3268059

[49] D'Angelo V, Crisci S, Casale F, Addeo R, Giuliano M, et al (2009) High Erk-1 activation and Gadd45a expression as prognostic markers in high risk pediatric haemolymphoproliferative diseases. J Exp Clin Cancer Res 28: 39 10.1186/1756-9966-28-39 19298651 PMC2664791

[50] Liebermann DA, Tront JS, Sha X, Mukherjee K, Mohamed-Hadley A, et al Gadd45 stress sensors in malignancy and leukemia. Crit Rev Oncog 16: 129–140. 22150313 10.1615/critrevoncog.v16.i1-2.120 PMC3268054

[51] Thalheimer FB, Wingert S, De Giacomo P, Haetscher N, Rehage M, et al Cytokine-regulated GADD45G induces differentiation and lineage selection in hematopoietic stem cells. Stem Cell Reports 3: 34–43. 10.1016/j.stemcr.2014.05.010 25068120 PMC4110750

[52] Puccetti E, Guller S, Orleth A, Bruggenolte N, Hoelzer D, et al (2000) BCR-ABL mediates arsenic trioxide-induced apoptosis independently of its aberrant kinase activity. Cancer Res 60: 3409–3413. 10910048

[53] Donnelly ML, Hughes LE, Luke G, Mendoza H, ten Dam E, et al (2001) The 'cleavage' activities of foot-and-mouth disease virus 2A site-directed mutants and naturally occurring '2A-like' sequences. J Gen Virol 82: 1027–1041. 11297677 10.1099/0022-1317-82-5-1027

[54] Beissert T, Puccetti E, Bianchini A, Guller S, Boehrer S, et al (2003) Targeting of the N-terminal coiled coil oligomerization interface of BCR interferes with the transformation potential of BCR-ABL and increases sensitivity to STI571. Blood 102: 2985–2993. 12829585 10.1182/blood-2003-03-0811

[55] Mian AA, Metodieva A, Najajreh Y, Ottmann OG, Mahajna J, et al (2012) p185(BCR/ABL) has a lower sensitivity than p210(BCR/ABL) to the allosteric inhibitor GNF-2 in Philadelphia chromosome-positive acute lymphatic leukemia. Haematologica 97: 251–257. 10.3324/haematol.2011.047191 22058195 PMC3269486

[56] Grignani F, Kinsella T, Mencarelli A, Valtieri M, Riganelli D, et al (1998) High-efficiency gene transfer and selection of human hematopoietic progenitor cells with a hybrid EBV/retroviral vector expressing the green fluorescence protein. Cancer Res 58: 14–19. 9426049

[57] Sternsdorf T, Puccetti E, Jensen K, Hoelzer D, Will H, et al (1999) PIC-1/SUMO-1-modified PML-retinoic acid receptor alpha mediates arsenic trioxide-induced apoptosis in acute promyelocytic leukemia. Mol Cell Biol 19: 5170–5178. 10373566 10.1128/mcb.19.7.5170 PMC84360

[58] R-Development-Core-Team (2005) R: A language and environment for statistical computing R Foundation for Statistical Computing, Vienna, Austria; http://www.R-project.org.ISBN3-900051-07-0.

[59] Gentleman RC, Carey VJ, Bates DM, Bolstad B, Dettling M, et al (2004) Bioconductor: open software development for computational biology and bioinformatics. Genome Biol 5: R80 15461798 10.1186/gb-2004-5-10-r80 PMC545600

[60] Bustin SA (2000) Absolute quantification of mRNA using real-time reverse transcription polymerase chain reaction assays. J Mol Endocrinol 25: 169–193. 11013345 10.1677/jme.0.0250169

