# Supplementary figures and images for: The Functional Interplay Between the t(9;22)-Associated Fusion Proteins BCR/ABL and ABL/BCR in Philadelphia Chromosome-Positive Acute Lymphatic Leukemia
Source: PLoS Genet. 2015 Apr 28;11(4):e1005144. doi: 10.1371/journal.pgen.1005144 (PMC4412790; doi:10.1371/journal.pgen.1005144)

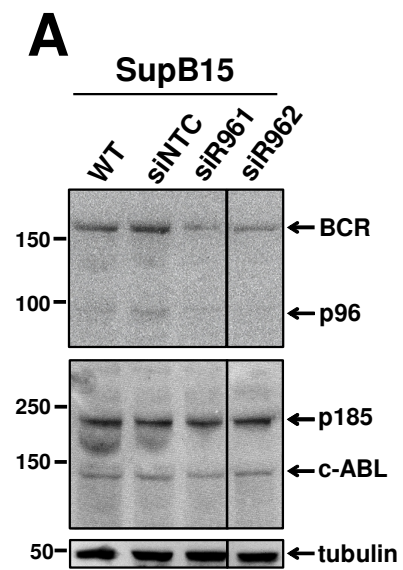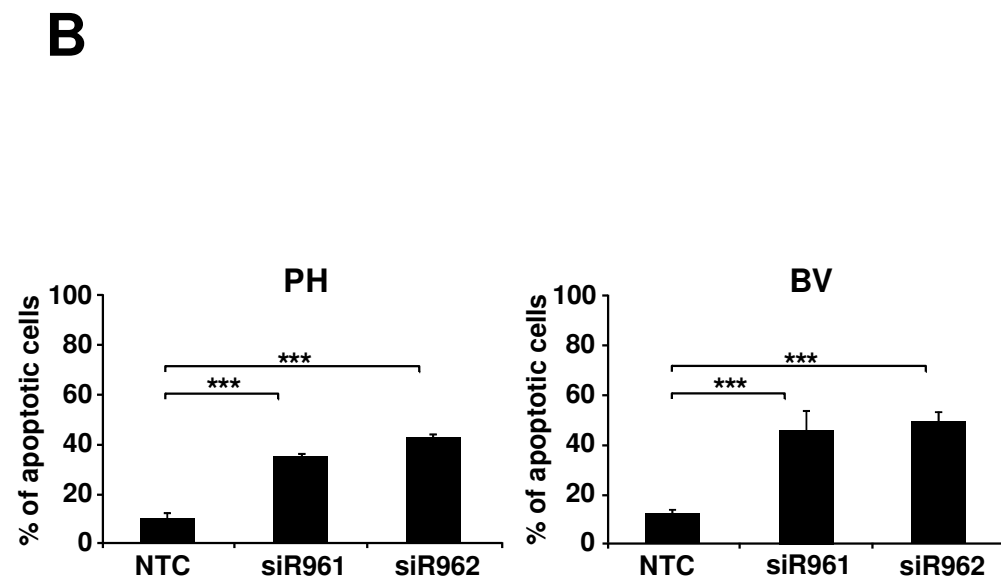

supplementary Figure 1

Supplement: S1 Fig — (A) SupB15 cells were lentivirally transduced with shRNAs (siR961 and siR962 and siNTC as control) against p96ABL/BCR. The effect on the expression of ABL/BCR and BCR/ABL, respectively, was revealed by using the indicated antibodies; anti-tubulin staining was used for loading control. (B) Induction of apoptosis. BV and PH cells were lentivirally transduced with shRNAs (siR961 and siR962 and siNTC as control) against p96ABL/BCR and apoptosis rate was measured using 7-AAD by FACS. One representative out of three experiments each performed in triplicates with similar results is given ± SD. (PDF) [file pgen.1005144.s001.pdf]

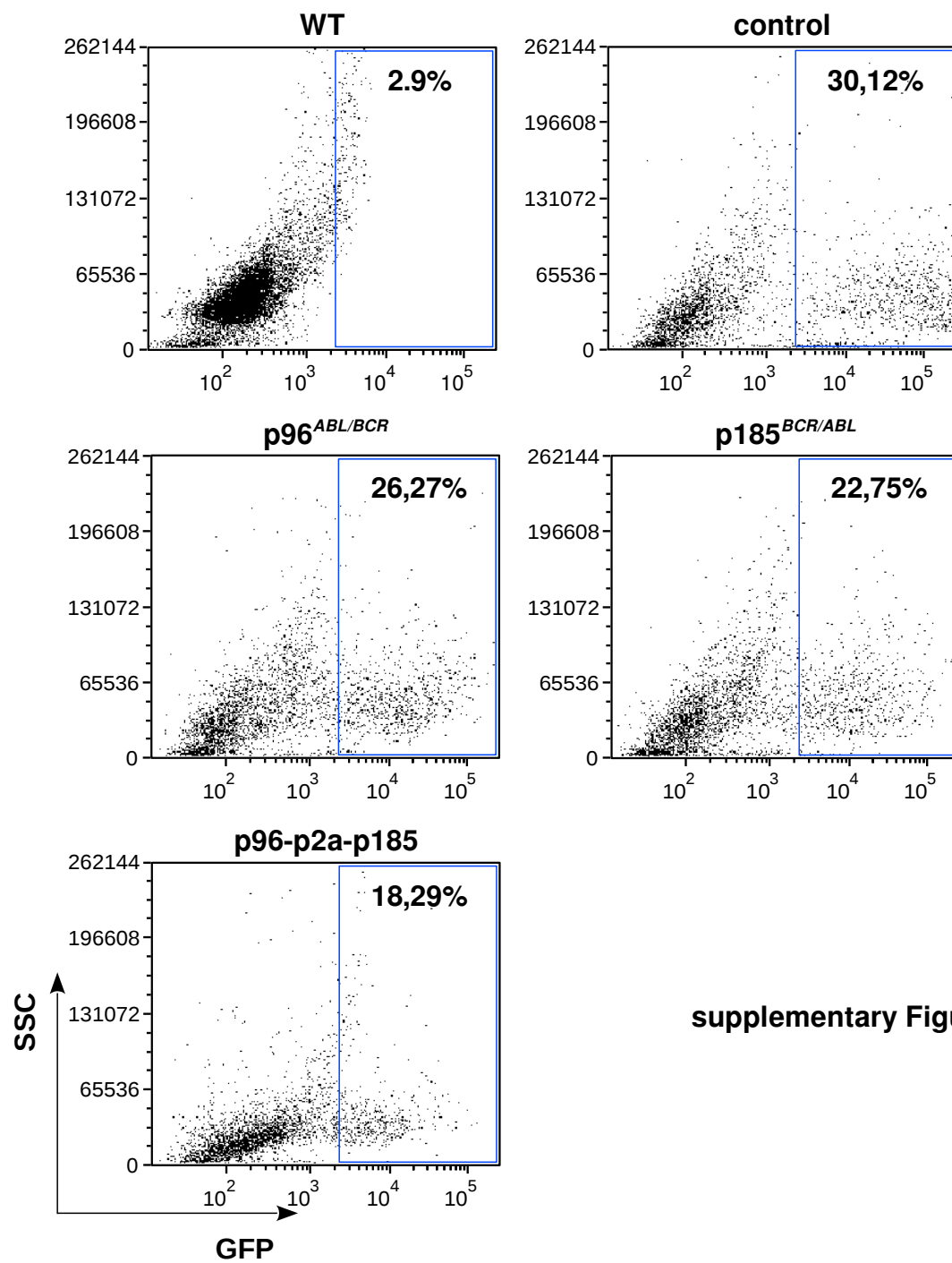

supplementary Figure 2

Supplement: S2 Fig — The expression of the transgenes used in this experiment was detected by FACS for the expression of GFP. The wild-type (WT) cells were taken as negative control for GFP expression. (PDF) [file pgen.1005144.s002.pdf]

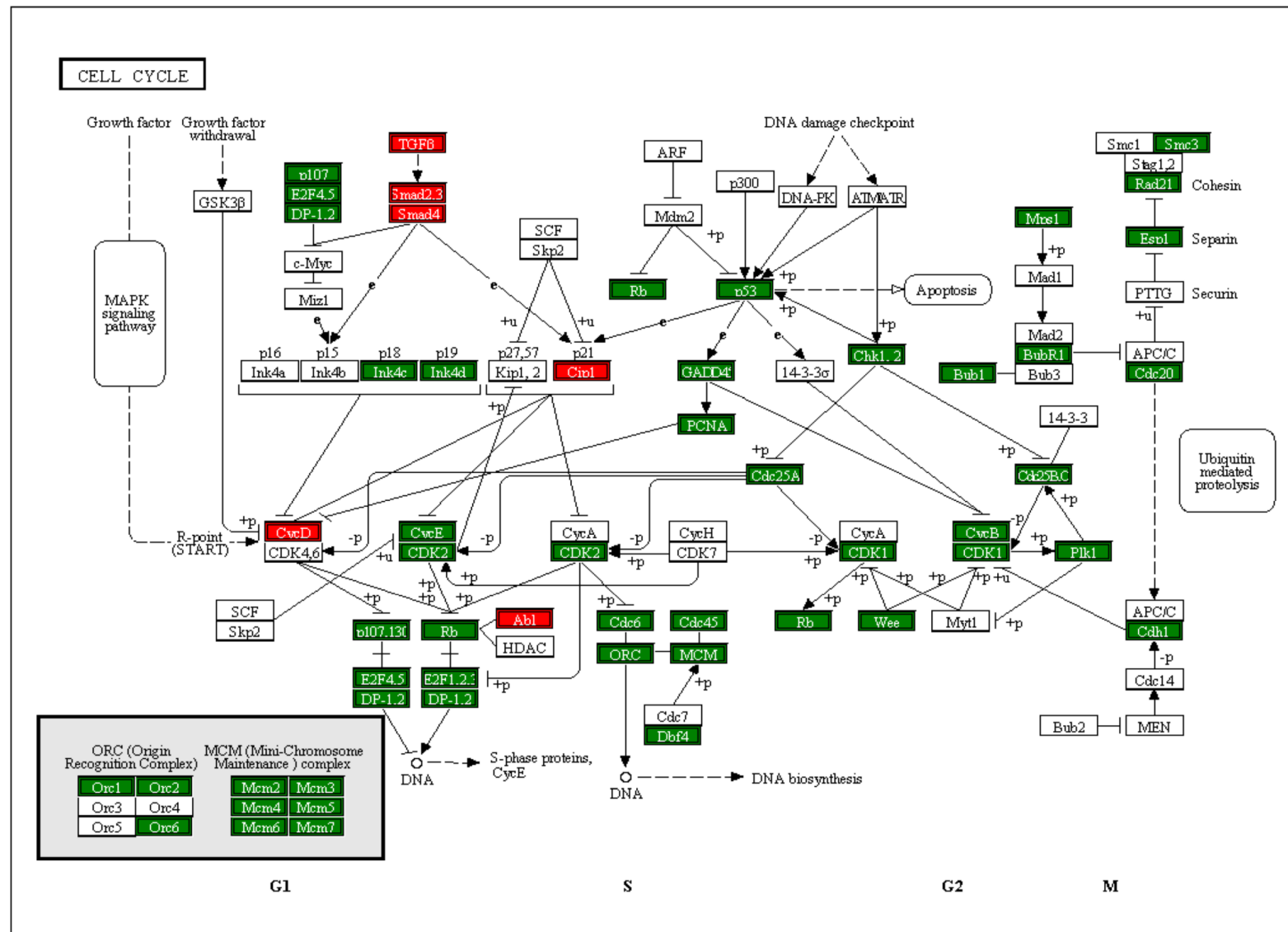

supplementary Figure 3

Supplement: S3 Fig — The cell cycle regulation genes and their interaction upon expression of the transgenes is visualized in this picture. The colored (green and red) genes are differentially regulated between p185BCR/ABL versus p96+p185 groups. The green colored genes are down-regulated (fold changes < -1) in p185BCR/ABL-containing spleens in comparison to spleens positive for both p96ABL/BCR and p185BCR/ABL. The red colored genes are up-regulated (fold changes > +2) in p185BCR/ABL in comparison to p96+p185 group. (PDF) [file pgen.1005144.s003.pdf]

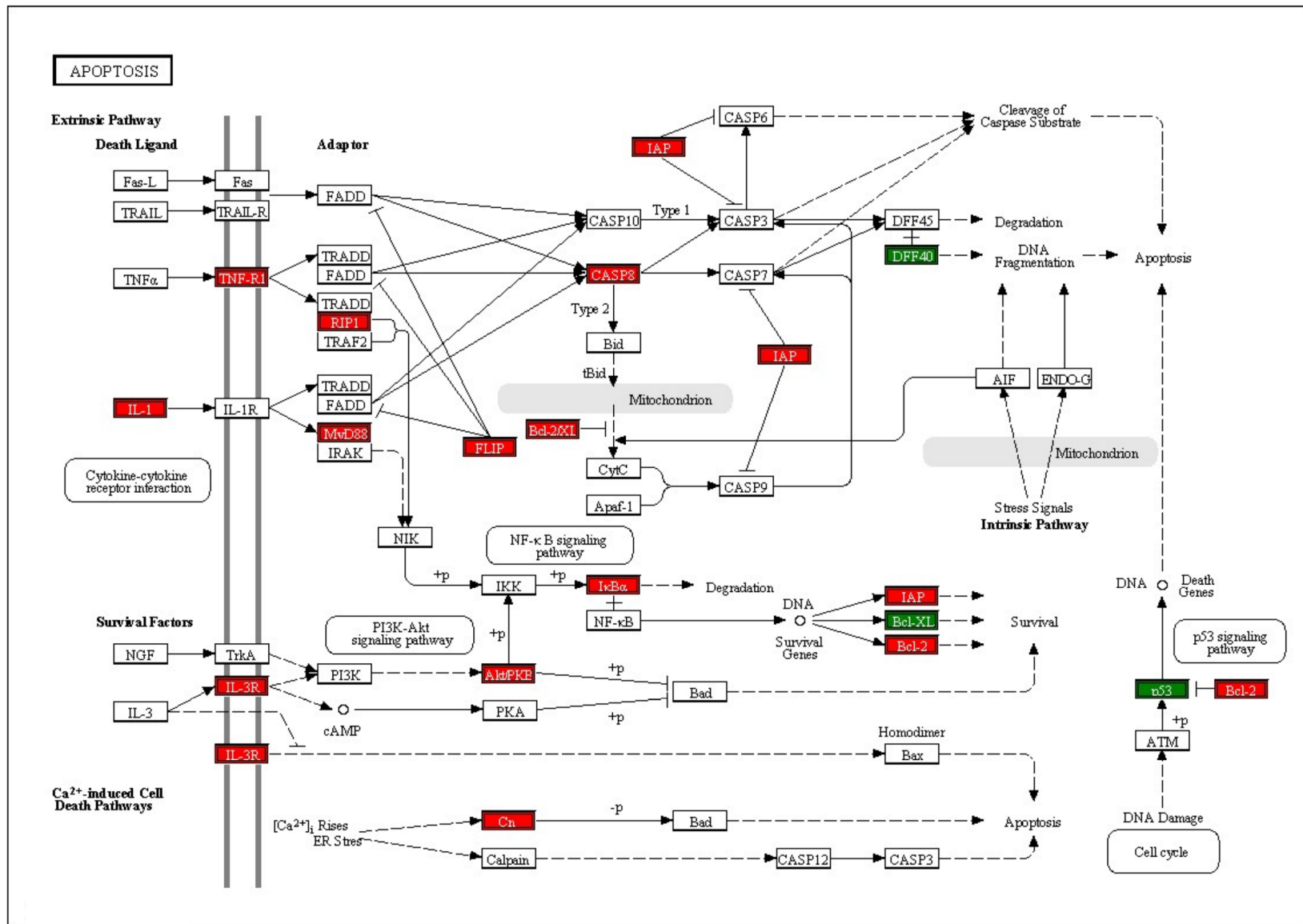

supplementary Figure 4

Supplement: S4 Fig — The genes related to apoptosis signaling and their interaction upon expression of the transgenes is visualized in this picture. The colored (green and red) genes are differentially regulated between p185BCR/ABL versus p96+p185 groups. The green colored genes are down-regulated (fold changes < -1) in p185BCR/ABL-containing spleens in comparison to spleens positive for both p96ABL/BCR and p185BCR/ABL. The red colored genes are up-regulated (fold changes > +2) in p185BCR/ABL in comparison to p96+p185 group. (PDF) [file pgen.1005144.s004.pdf]

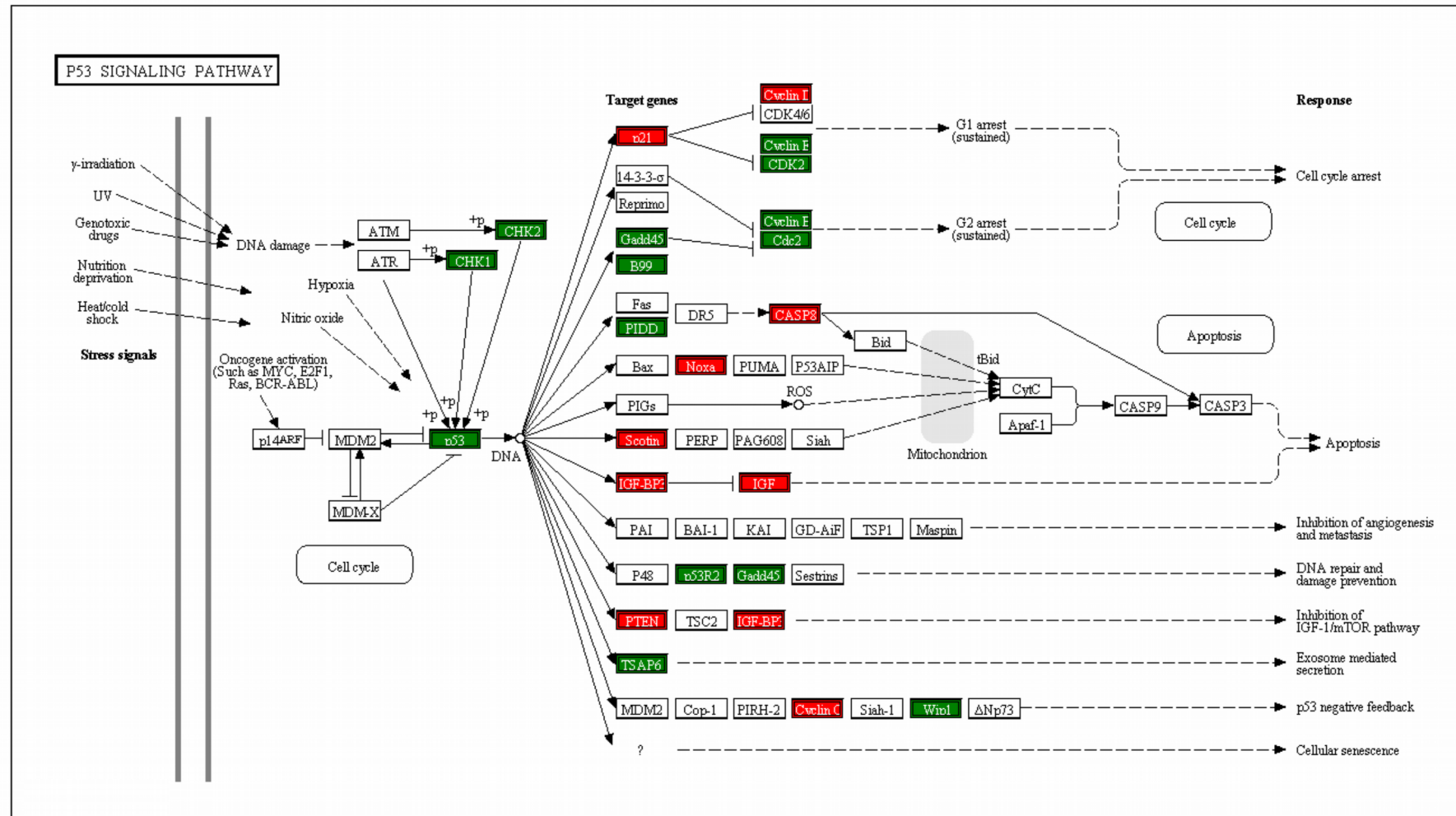

supplementary Figure 5

Supplement: S5 Fig — The genes related to p53 pathway and their interaction upon expression of the transgenes is visualized in this picture. The colored (green and red) genes are differentially regulated between p185BCR/ABL versus p96+p185 groups. The green colored genes are down-regulated (fold changes < -1) in p185BCR/ABL-containing spleens in comparison to the spleen which contained both p96ABL/BCR and p185BCR/ABL. The red colored genes are up-regulated (fold changes > +2) in p185BCR/ABL in comparison to p96+p185 group. (PDF) [file pgen.1005144.s005.pdf]

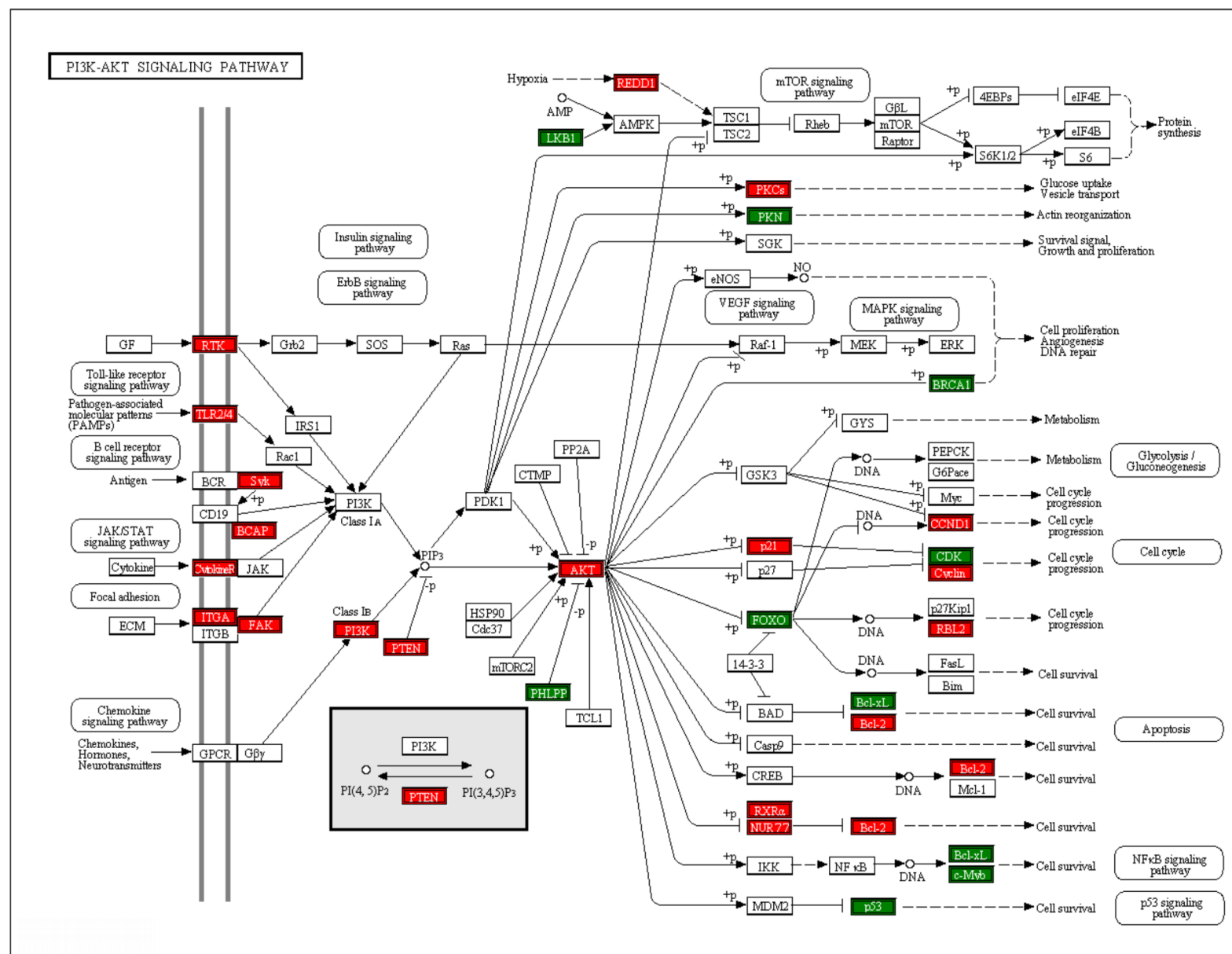

supplementary Figure 6

Supplement: S6 Fig — The genes related to PI3K/AKT pathway and their interaction upon expression of the transgenes is visualized in this picture. The colored (green and red) genes are differentially regulated between p185BCR/ABL versus p96+p185 group. The green colored genes are down-regulated (fold changes < -1) in p185BCR/ABL- containing spleens in comparison to the spleens which contained both p96ABL/BCR and p185BCR/ABL. The red colored genes are up-regulated (fold changes > +2) in p185BCR/ABL in comparison to p96+p185 group. (PDF) [file pgen.1005144.s006.pdf]

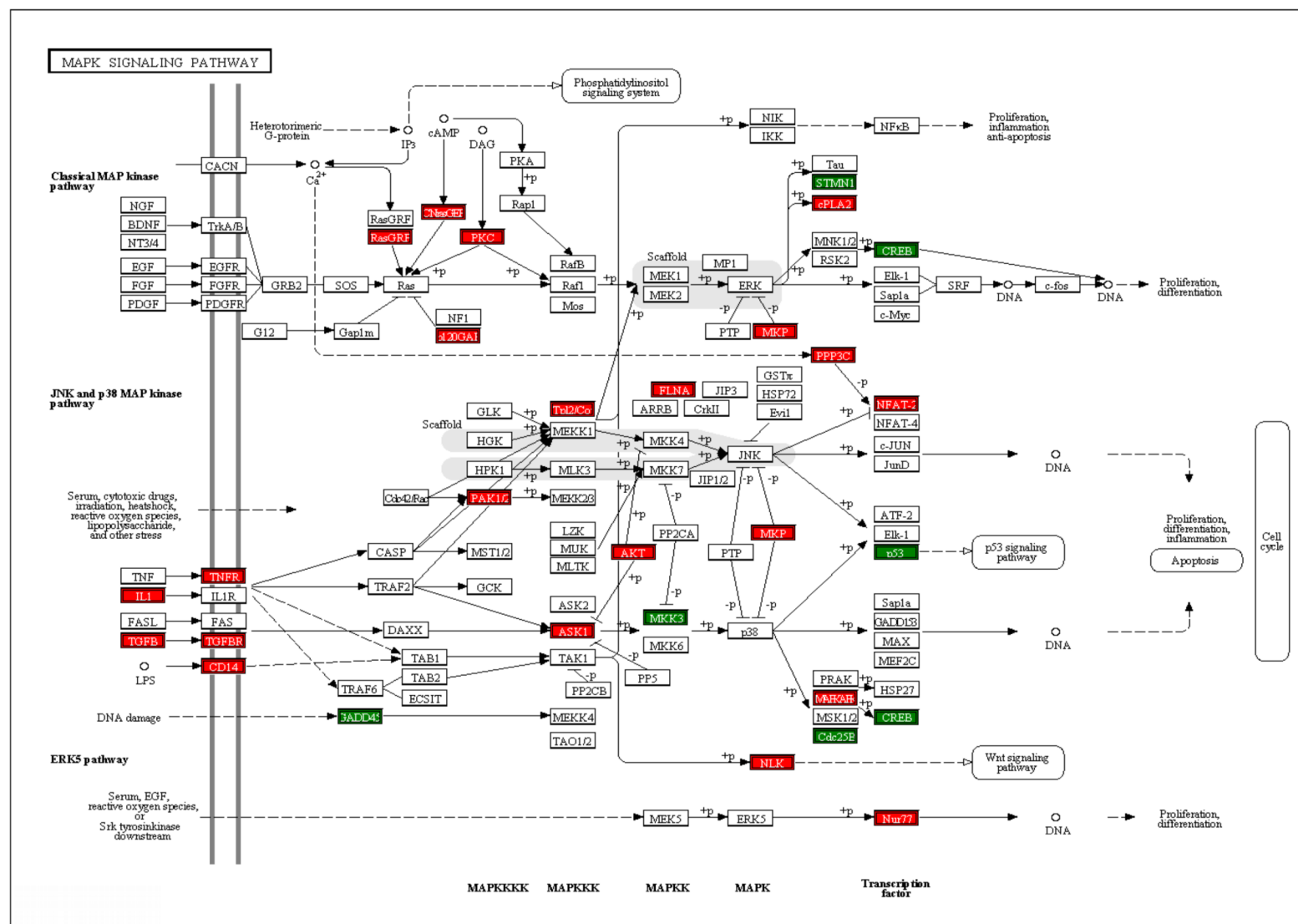

Supplement: S7 Fig — The genes related to MAP-kinase signaling and their interaction upon expression of the transgenes is visualized in this picture. The colored (green and red) genes are differentially regulated between p185BCR/ABL versus p96+p185 groups. The green colored genes are down-regulated (fold changes < -1) in p185BCR/ABL-containing spleens in comparison to the spleens positive for both p96ABL/BCR and p185BCR/ABL. The red colored genes are up-regulated (fold changes > +2) in p185BCR/ABL in comparison to p96+p185 group. (PDF) [file pgen.1005144.s007.pdf]

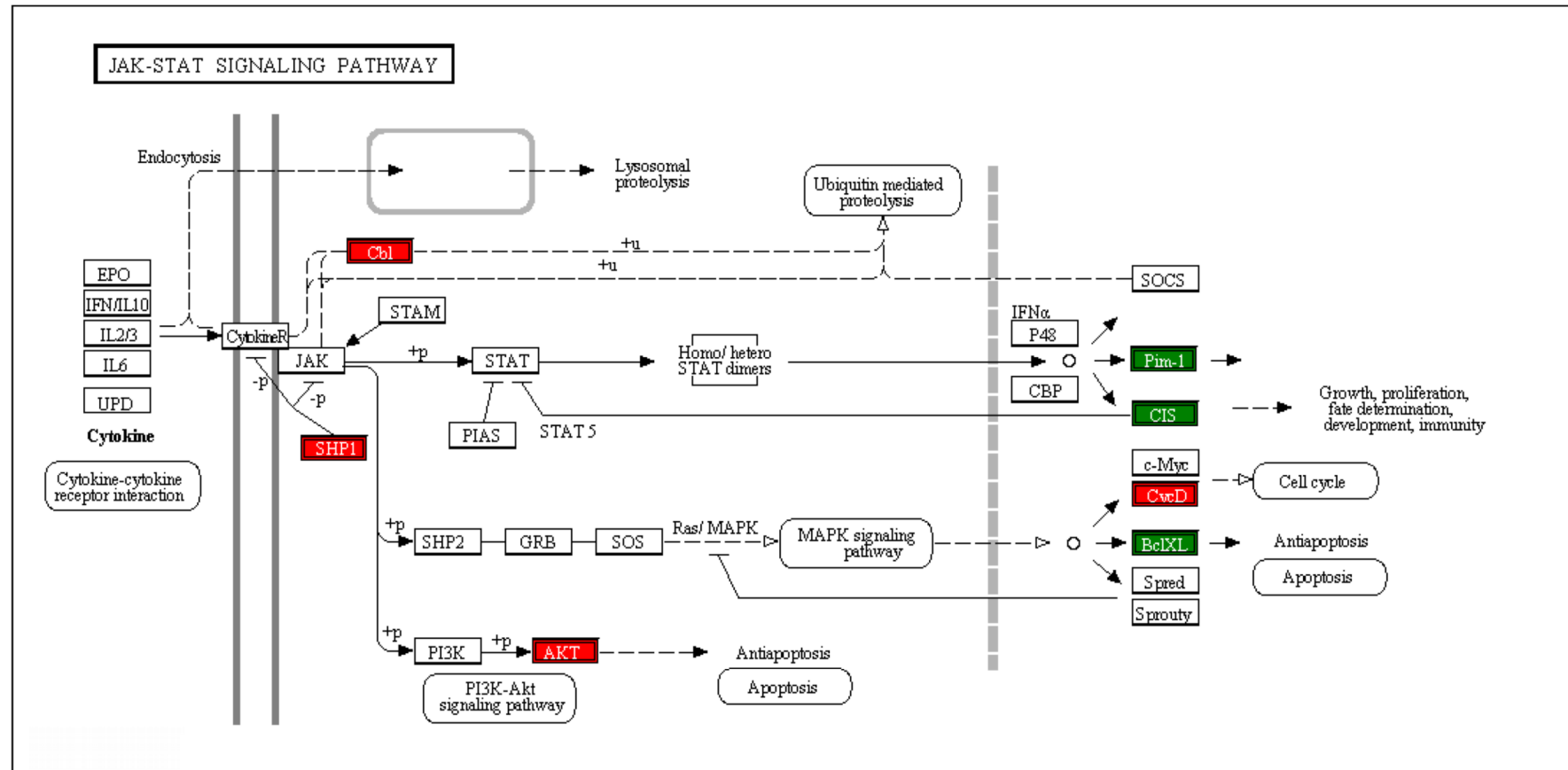

supplementary Figure 8

Supplement: S8 Fig — The genes related to JAK-STAT signaling and their interaction upon expression of the transgenes is visualized in this picture. The colored (green and red) genes are differentially regulated between p185BCR/ABL versus p96+p185 groups. The green colored genes are down-regulated (fold changes < -1) in p185BCR/ABL-containing spleens in comparison to the spleens positive for both p96ABL/BCR and p185BCR/ABL. The red colored genes are up-regulated (fold changes > +2) in p185BCR/ABL in comparison to p96+p185 group. (PDF) [file pgen.1005144.s008.pdf]

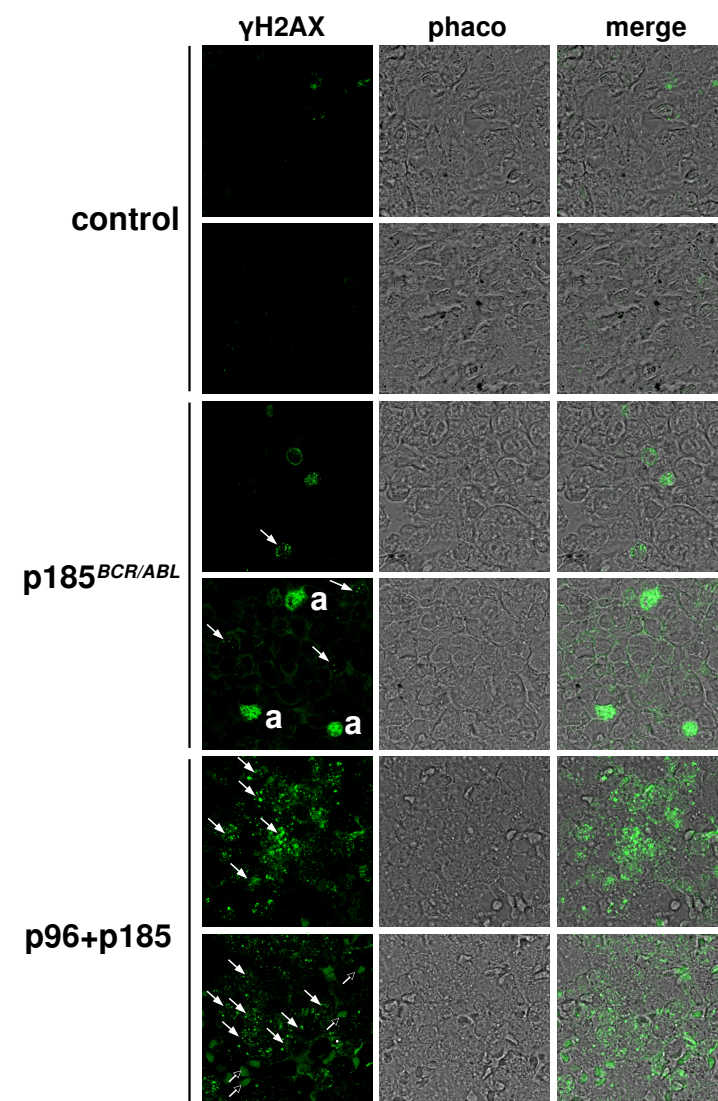

supplementary Figure 9

Supplement: S9 Fig — Green fluorochrome—γH2AX; phaco—phase contrast. Control—empty vector transduced control spleen. p185—p185BCR/ABL- positive leukemia; p185+p96—p96ABL/BCR-p185BCR/ABL-positive leukemia. (PDF) [file pgen.1005144.s009.pdf]

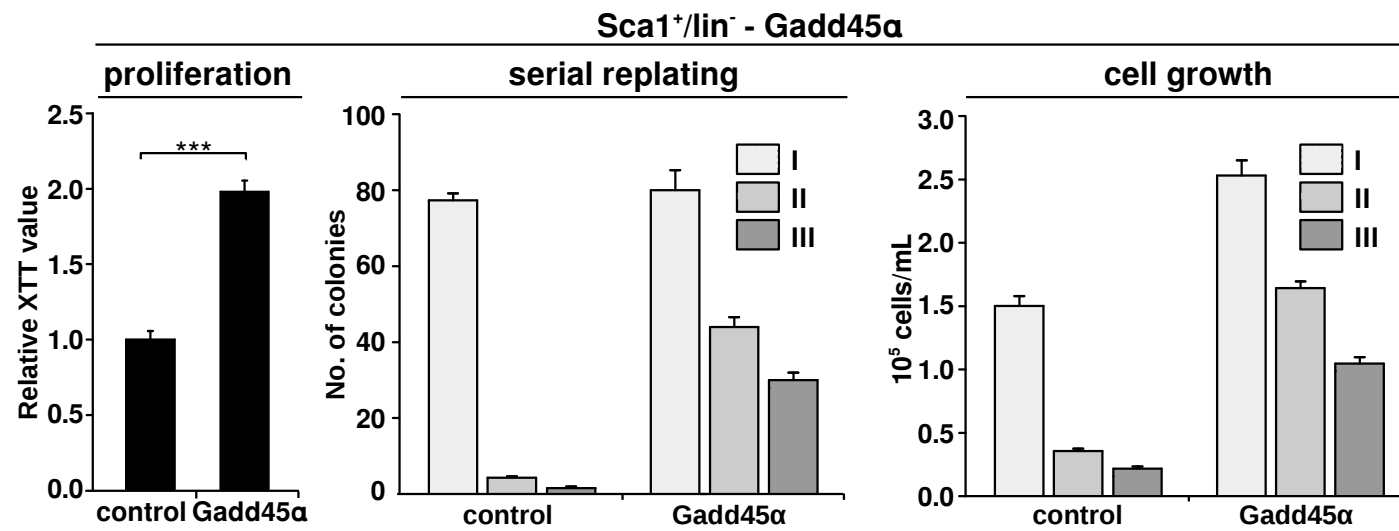

supplementary Figure 10

Supplement: S10 Fig — Sca1+/lin- cells were immunomagnetically isolated from murine BM and the cells were transduced with empty virus or with Gadd45α plated either in liquid culture or in semi-solid medium supplemented with growth factors for determination of the proliferation and serial replating potential, respectively. Proliferation was assessed by XTT at day 5 after plating. Colony numbers were counted on day 10, cells were harvested and serially replated. Each time cells were counted for the determination of cell growth (I-IV-plating rounds). (PDF) [file pgen.1005144.s010.pdf]
