# Supplementary material for: The Functional Interplay Between the t(9;22)-Associated Fusion Proteins BCR/ABL and ABL/BCR in Philadelphia Chromosome-Positive Acute Lymphatic Leukemia
Source: PLoS Genet. 2015 Apr 28;11(4):e1005144. doi: 10.1371/journal.pgen.1005144 (PMC4412790; doi:10.1371/journal.pgen.1005144)
Supplement: S1 Text — (DOCX) [file pgen.1005144.s011.docx]

**S1_Text**

**Microarray preprocessing**

**Normalization.** Probe level normalization was conducted using the variance stabilization method ^1^. This method renders the variance of probe intensities approximately independent of their expected expression levels. Parameters (offset and a scaling factor) are estimated for each microarray, in consideration of the fact that a fair fraction of probes is not differentially expressed across the samples. In view of computational complexity of the algorithm parameters are estimated on a random subset of probes and are then used to transform the complete arrays.

**Probeset summary.** Probeset summarization was calculated using the medial polish method ^2^ on the normalized data. For each Probeset a robust additive model was fitted across the arrays, considering the different sensitivity of the Probeset via the probe effect.

**Heatmap.** A heatmap is a two dimensional plot, where the same signals display with the same colors. It is not only a visually impression of the gene expression. Heatmaps are done with the Spotfire software (Spotfire Decision Site 9.1.2).

**Differentially expression.** Many of the genes on the microarray won’t be expressed, or might have only a small variability across the samples. First an expression intensity filter is used to reduce the dimension of the microarray data: The data are filtered with an intensity (the intensity of a gene should be above 100 in at least 0.25 percent of the samples, if the group size is equal) and a variance filter (the interquartile range of log2 intensities should be at least 0.5, if the groups size is equal). After the expression intensity filtering p values were calculated with two sample t-test (variance=equal) to identify genes that are differentially expressed between two gro

ups. For the multiple testing problems a False Discovery Rate (FDR) ^3^ was used. Also Fold changes (FC) between the two groups was calculated for each gene. The lists of differentially expressed genes were filtered with FDR and FC criteria.

**Supplementary Material and Methods**

**Immunofluorescence - confocal laser scan analysis of formalin-fixed paraffin embedded (FFPE) tissues sections**

Spleen sections of 4 µm thickness were dewaxed in xylene 2 times for 10 min each and re-hydrated in graded alcohol series. Epitope retrieval was performed in a water bath at 60°C over night in 10 mM HIER citrate buffer pH 6 (Zytomed Systems, Germany). Unspecific binding was blocked with 5% non-fat dry milk (Carl Roth, Germany) in TBS for 60 min. Primary antibody Υ-H2AX (pSer9) (mouse monoclonal, Biolegend) was diluted 1:100 in antibody solution (1% Milk and 0.2% TritonX-100 in TBS). The sections were incubated 60 min at RT followed by extensive washing in TBS and incubation for 45 min. at RT with Alexa Fluor® 488 anti-mouse Ig antibodies (Life Technologies, Darmstadt, Germany). For the confocal laser scan analysis images were acquired by a Leica TCS-SP5 system (Leica, Wetzlar, Germany) under identical conditions for pinhole opening, laser power, photomultiplier tension and layer number. During data elaboration by Fiji software (www.fiji.sc) identical parameters were applied for all samples.

**Lentiviral expression vector - Gadd45α**

The ORF of murine Gadd45α was amplified by RT-PCR (Gaa-fw 5´-ttg gcc ggc cga ggg act cgc act tgc aat atg-3´; Gaa-rev 5´-tta cta gtt gaa ctc ggc ccc ttg aca t-3´) and cloned into the pRRL.PPT.SF - FT IRES-VENUSnucmemprev already described elsewhere ^4^.

**Supplementary references**

1. Huber W, von Heydebreck A, Sultmann H, et al. Variance stabilization applied to microarray data calibration and to the quantification of differential expression. Bioinformatics*.* 2002;18 Suppl 1:S96-104.

2. Tukey JW. Exploratory Data analysis. Addison-Wesley, Reading Masschusetts, USA*.* 1977.

3. Hochberg Y, Benjamini Y. More powerful procedures for multiple significance testing. Stat Med*.* 1990;9:811-818.

4. Thalheimer FB, Wingert S, De Giacomo P, Haetscher N, Rehage M, et al. Cytokine-regulated GADD45G induces differentiation and lineage selection in hematopoietic stem cells. Stem Cell Reports 3: 34-43.
